# The osteohistology of gorgonopsian therapsids and implications for Permo‐Triassic theriodont growth

**DOI:** 10.1111/joa.14201

**Published:** 2024-12-20

**Authors:** Jennifer Botha

**Affiliations:** ^1^ Evolutionary Studies Institute, University of the Witwatersrand Johannesburg South Africa; ^2^ GENUS: DSTI‐NRF Centre of Excellence in Palaeosciences University of the Witwatersrand Johannesburg South Africa

**Keywords:** bone histology, cynodont, gorgonopsia, life history, Permian, therapsid, therocephalian

## Abstract

During the Late Permian, saber‐toothed gorgonopsian therapsids were the dominant terrestrial predators, playing crucial roles as apex predators alongside therocephalian therapsids within Permian terrestrial ecosystems. The entire gorgonopsian clade went extinct during the Permo‐Triassic mass extinction, leaving other therapsids to continue into the Triassic. Gorgonopsians have not been well studied, particularly in terms of their growth patterns, with only a few genera having undergone osteohistological analysis. In this study, I present a thorough osteohistological examination of the most extensive collection of gorgonopsian specimens to date, spanning a diverse range of limb bones sourced from various species. The osteohistological analysis of gorgonopsian specimens reveals a trend of rapid growth characterized by a highly vascularized woven‐parallel complex. The abundance of growth marks and variable zone widths suggests a growth trajectory that could indicate longer lifespans and slower growth rates when compared to Early Triassic therapsids. The high vascularity, coupled with the observed growth patterns, implies that gorgonopsians experienced rapid growth during favorable conditions. However, the multiple growth marks indicate that they likely had the capacity for longer lifespans and more gradual maturation than their Early Triassic counterparts. Additionally, their ability to reach later ontogenetic stages supports the hypothesis that favorable environmental conditions facilitated larger body sizes. In contrast, Early Triassic therapsids primarily consisted of juveniles or individuals who reached reproductive maturity within a year, likely indicative of harsher conditions that contributed to higher mortality rates at younger ages. The onset of decreased growth rates, usually indicative of reproductive maturity, occurred later in gorgonopsians compared to Early Triassic therapsids and may have contributed to their decline, as the heightened juvenile mortality rates during the PTME would have limited the gorgonopsians' ability to reproduce effectively.

## Graphical abstract

Permian gorgonopsian therapists had rapid, annually interrupted growth and show longer lifespans than early Triassic therapists.

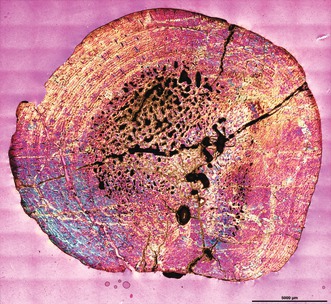

## 1 INTRODUCTION

During the Late Permian, saber‐toothed gorgonopsians were the dominant terrestrial predators (Broom, 1932; Gebauer, 2007; Kemp, 2005; Norton, 2012). Gorgonopsians belong to a lineage of theriodont therapsids, encompassing gorgonopsians, therocephalians, and non‐mammaliaform cynodonts (herein referred to as cynodonts), which eventually gave rise to mammals by the end of the Triassic Period. Gorgonopsians, alongside therocephalians, occupied crucial roles as apex predators within Permian terrestrial ecosystems. Gorgonopsians exhibited significant variation in body size, with smaller species such as *Eriphostoma*, *Cyonosaurus*, and *Aelurosaurus* having basal skull lengths of approximately 15 cm, whereas larger species such as *Inostrancevia*, *Dinogorgon*, and *Rubidgea* had basal skull lengths up to 60 cm (Angielczyk & Kammerer, 2018).

Primarily found in South African and Russian strata, gorgonopsians have also been recovered from deposits in Malawi, Niger, Tanzania, and Zambia (Brant & Sidor, 2023; Gebauer, 2007; Sigogneau, 1970; Sigogneau‐Russell, 1989; Smiley et al., 2008). They are characterized by “a transverse lamina of the septomaxilla separating the external nares into two compartments; a steep, highly elevated mandibular symphysis; a large, anteriorly curved retroarticular process; and a cruciate pattern of ridges on the surface of the reflected lamina of the angula” (Angielczyk & Kammerer, 2018). These predators were specialized for hunting large prey, with adaptations such as hyper‐elongated canines, serrated incisors, and a jaw mechanism that allowed them to open their mouths nearly 90 degrees (Kemp, 1969, 1982).

Despite their abundance in South African Karoo collections, gorgonopsians remain less understood compared to other theriodonts due to longstanding issues regarding their taxonomy. Gorgonopsian skulls are relatively conserved, complicating the identification of subclades. However, there is consensus on the existence of a distinct Russian subclade, Inostranceviinae, and an African subclade, Rubidgeinae (Angielczyk & Kammerer, 2018). The recent discovery of the traditionally Russian *Inostrancevia* in the uppermost Permian deposits of the South African Karoo Basin indicates potential migration between the Laurasian and Gondwanan regions, further complicating the understanding of their distribution (Kammerer et al., 2023). Importantly, the entire gorgonopsian clade went extinct during the Permo‐Triassic mass extinction (PTME) approximately 252 million years ago, leaving therocephalians, cynodonts, and the non‐theriodont therapsid anomodonts to continue into the Triassic (Viglietti et al., 2021).

Whereas therocephalians and cynodonts have been extensively studied for decades due to their close relationship with the mammalian lineage, gorgonopsians have remained relatively elusive, leaving much of their paleobiology unknown, including their growth patterns. Osteohistological analysis can be used to understand the life history of extinct vertebrates, a method that has shed considerable light on the growth dynamics of therocephalians and cynodonts. de Ricqlès (1969) first examined a femur identified as *Aelurognathus* and described the bone tissue as highly vascularized fibrolamellar bone. Decades later, Ray et al. (2004) and Chinsamy‐Turan and Ray (2012) studied the same two specimens SAM‐PK‐K10000 and SAM‐PK‐10188 identified as *Aelurognathus* and *Scylacops*, respectively. However, *Scylacops* is now considered a junior synonym of *Aelurognathus* (Gebauer, 2007). Botha‐Brink et al. (2016) gave a further brief description of SAM‐PK‐K10428 identified as the small‐bodied *Cyonosaurus*. Apart from these two genera, and an indeterminate gorgonopsian with a pathological condition (Kato et al., 2020), no gorgonopsian material has undergone paleohistological analysis.

However, the recent research by authors such as Gebauer, Norton, and Kammerer has clarified aspects of the alpha taxonomy, enabling further exploration beyond taxonomy itself.

Given the importance of gorgonopsians as apex predators in Permian terrestrial ecosystems and their close relationship with therocephalians and cynodonts, it is crucial to understand their biology. This knowledge is particularly important because the reasons for their extinction during the PTME remain poorly understood. Studying the factors behind the differential survival of species during past mass extinctions can provide valuable insights for predicting and mitigating future extinction events.

This study aims to test several key hypotheses regarding gorgonopsian growth and life history (1) to determine whether gorgonopsians had a longer lifespan and slower growth rates compared to Early Triassic therapsids, (2) to investigate how environmental conditions during the Permian supported larger body sizes and extended ontogenetic stages in gorgonopsians, (3) to evaluate the significance of subadult dominance in the gorgonopsian fossil record as an indicator of a stable environment conducive to growth, (4) to assess whether gorgonopsians demonstrated a growth strategy allowing for reproductive maturity at larger sizes relative to Early Triassic therapsids, and (5) to analyze growth and mortality patterns in gorgonopsians during the transition to the Triassic, particularly in the context of the PTME.

## 2 MATERIALS AND METHODS

### 2.1 Material

The study material comprises 18 specimens (Table 1), some of which had been identified to genus level on various South African databases. However, upon further examination by an expert (Christian Kammerer, pers. comm. 2023), some of the generic identifications were updated, leaving six specimens positively identified, a possible *Tigricephalus* and a possible new species. All postcranial material was associated with skull material, allowing for positive identification, or hopefully, with the recovery of more specimens, future generic identifications of those specimens that have been labeled as indeterminate in this study. Those that have not been positively identified to genus level have been left as “indeterminate,” but it is clear from the osteohistological analysis that various specimens can be distinguished as different genera (see results and detailed osteohistological description in Data S1). SAM‐PK‐10000, previously identified as *Aelurognathus* (Chinsamy‐Turan & Ray, 2012; Ray et al., 2004), is now considered indeterminate (Kammerer et al., 2023). The material comes from the Iziko South African Museum, Cape Town, the National Museum, Bloemfontein, the Evolutionary Studies Institute, Johannesburg, and the Council for Geoscience, Pretoria. Several of the specimens include multiple elements (49 in the total sample) allowing for growth curves to be estimated. Complete transverse sections of each bone are available on Morphosource under project ID 000668546.

**TABLE 1:** List of gorgonopsian materials used in this study. AZ, Assemblage Zone.

Accession number	Identification	Element	Locality	AZone
SAM‐PK‐K10428b	*Cyonosaurus*	Ulna	Doornplaats, Graaff‐Reinet	*Daptocephalus*
SAM‐PK‐10188a	*Cyonosaurus*	Humerus	Quagga Fontein 82, Dunedin, Beaufort West	*Cistecephalus*
SAM‐PK‐10188b	*Cyonosaurus*	Femur	Quagga Fontein 82, Dunedin, Beaufort West	*Cistecephalus*
SAM‐PK‐10188c	*Cyonosaurus*	Radius	Quagga Fontein 82, Dunedin, Beaufort West	*Cistecephalus*
SAM‐PK‐10188d	*Cyonosaurus*	Ulna	Quagga Fontein 82, Dunedin, Beaufort West	*Cistecephalus*
SAM‐PK‐10188e	*Cyonosaurus*	Tibia	Quagga Fontein 82, Dunedin, Beaufort West	*Cistecephalus*
SAM‐PK‐10188f	*Cyonosaurus*	Rib	Quagga Fontein 82, Dunedin, Beaufort West	*Cistecephalus*
SAM‐PK‐K10000a	Indeterminate	Humerus	Leeuw Kloof 43, Leeuwkloof, Beaufort West	*Endothiodon*
SAM‐PK‐K10000b	Indeterminate	Radius	Leeuw Kloof 43, Leeuwkloof, Beaufort West	*Endothiodon*
SAM‐PK‐K10000c	Indeterminate	Ulna	Leeuw Kloof 43, Leeuwkloof, Beaufort West	*Endothiodon*
SAM‐PK‐K10000d	Indeterminate	Tibia	Leeuw Kloof 43, Leeuwkloof, Beaufort West	*Endothiodon*
SAM‐PK‐K4460	*Gorgonops torvus*	Humerus	Melton Wold 158, Melton Wold Victoria West	*Tapinocephalus/Endothiodon*
SAM‐PK‐K10035a	*Aelurognathus tigriceps*	Humerus	Walplaats 1, Walplaats, Aberdeen	*Daptocephalus*
SAM‐PK‐K10035b	*Aelurognathus tigriceps*	Ulna	Walplaats 1, Walplaats, Aberdeen	*Daptocephalus*
SAM‐PK‐K10110	New species?	Humerus	Walplaats 1, Walplaats, Aberdeen	*Daptocephalus*
SAM‐PK‐K8622a	Indeterminate	Femur	Rust 126, Doornplaats, Graaff‐Reinet	*Daptocephalus*
SAM‐PK‐K8622b	Indeterminate	Tibia	Rust 126, Doornplaats, Graaff‐Reinet	*Daptocephalus*
SAM‐PK‐K8622c	Indeterminate	Fibula	Rust 126, Doornplaats, Graaff‐Reinet	*Daptocephalus*
SAM‐PK‐K8623a	Indeterminate	Humerus	Rust 126, Doornplaats, Graaff‐Reinet	*Daptocephalus*
SAM‐PK‐K8623b	Indeterminate	Ulna	Rust 126, Doornplaats, Graaff‐Reinet	*Daptocephalus*
Sam‐pk‐k8623c	Indeterminate	Radius	Rust 126, Doornplaats, Graaff‐Reinet	*Daptocephalus*
SAM‐PK‐K8623d	Indeterminate	Femur	Rust 126, Doornplaats, Graaff‐Reinet	*Daptocephalus*
SAM‐PK‐K10622c	Indeterminate	Femur	Lossekop 4, Loskop, Murraysburg	*Endothiodon*
SAM‐PK‐K10622d	Indeterminate	Femur	Lossekop 4, Loskop, Murraysburg	*Endothiodon*
CGS AF 391‐83a	Indeterminate	Humerus	Unknown	Unknown
CGS AF 391‐83b	Indeterminate	Radius	Unknown	Unknown
CGS AF 391‐83c	Indeterminate	Ulna	Unknown	Unknown
CGS AF 391‐83d	Indeterminate	Femur	Unknown	Unknown
CGS FL‐43a	Indeterminate	Humerus	Unknown	Unknown
CGS FL‐43b	Indeterminate	Radius right	Unknown	Unknown
CGS FL‐43c	Indeterminate	Ulna right	Unknown	Unknown
CGS FL‐43d	Indeterminate	Femur	Unknown	Unknown
CGS FL‐43e	Indeterminate	Tibia right	Unknown	Unknown
CGS FL‐43f	Indeterminate	Ulna left	Unknown	Unknown
CGS FL‐43 g	Indeterminate	Radius left	Unknown	Unknown
CGS FL‐43 h	Indeterminate	Tibia left	Unknown	Unknown
BP/1/4940	Indeterminate	Humerus	Graaff‐Reinet townlands	*Cistecephalus*
SAM‐PK‐K6415a	*Tigricephalus kingwilli*	Radius	Adjoining Quagga Fontein 83, Beaufort West	*Cistecephalus?*
SAM‐PK‐K6415b	*Tigricephalus kingwilli*	Ulna	Adjoining Quagga Fontein 83, Beaufort West	*Cistecephalus?*
SAM‐PK‐K6407a	Indeterminate	Humerus	Unknown	Unknown
SAM‐PK‐K6407b	Indeterminate	Femur	Unknown	Unknown
BP/1/4259a	Indeterminate	Humerus	Oudeberg Pass, Graaff‐Reinet	*Cistecephalus*
BP/1/4259b	Indeterminate	Radius	Oudeberg Pass, Graaff‐Reinet	*Cistecephalus*
BP/1/4259c	Indeterminate	Ulna	Oudeberg Pass, Graaff‐Reinet	*Cistecephalus*
BP/1/1258a	Indeterminate	Humerus	Geitjies Kop, Graaff‐Reinet	*Cistecephalus*
BP/1/1258b	Indeterminate	Radius	Geitjies Kop, Graaff‐Reinet	*Cistecephalus*
BP/1/1258c	Indeterminate	Ulna	Geitjies Kop, Graaff‐Reinet	*Cistecephalus*
BP/1/1533	*Arctops willistoni*	Humerus	Rooipoort, Murraysburg	*Cistecephalus*
NMQR 4000	*Inostrancevia africana*	Femur	Nooitgedacht 68, Bethulie	*Daptocephalus*

### 2.2 Methods

Limb bones were preferentially chosen as the midshaft of these bones preserves the longest growth record of an individual. One rib from specimen SAM‐PK‐10188 was included. New sections were created for the already published SAM‐PK‐K10000 and SAM‐PK‐10188. All elements were photographed, measured, and cast prior to thin sectioning. Thin sectioning was completed at the Evolutionary Studies Institute, University of the Witwatersrand, Johannesburg following standard procedures (see Botha‐Brink et al., 2018 for detailed methods). A detailed osteohistological description of each element of every specimen sampled is presented in the Data S1 and Figures S1–, S10.

The distance between annual growth marks was measured in microns using NIS Elements 4.5 (Data S2, Table S1). Unfortunately, growth marks could not always be traced around entire sections, preventing the estimation of each growth mark's circumference. Thus, the thickest part of the cortex of each element, representing the maximum growth rate, was measured for each bone (similar to Chapelle et al., 2021). Maximum growth rates (amount of microns within a zone, a zone being defined as the region of bone tissue deposition between growth marks) were based on the amount of bone deposition between the first and second growth marks because the first zone varied depending on the size and shape of the medullary cavity. Thus, the first complete zone was used to compare growth rates between individuals (however, data are provided for the period between the medullary cavity border and the first growth mark in Table S1). Complete digitally rendered cross‐sections were used to create black (representing bone) and white (representing space) images and put through the program Bone Profiler (Girondot & Laurin, 2003) in order to calculate several bone compactness parameters: the compactness of the cortex (Cg, global compactness), bone wall thickness (CDI, corticodiaphyseal index), S (width of the corticomedullary transition zone), and P (the extent of the medullary cavity) (Data S2, Table S2). The width between growth marks and compactness parameters were also calculated for several similar‐sized therocephalians (*Lycosuchus* and a scylacosaurid) and cynodonts (*Cynognathus* and *Diademodon*) for comparison (Data S2, Table S1). Binary images were only made for elements that had not been crushed and where the cross‐sections had been taken from the middle of the midshaft. Osteohistology terminology follows that of Prondvai et al. (2014) and de Buffrénil and Quilhac (2021).

## 3 RESULTS

### 3.1 Bone tissue patterns

Below is a summary of the bone tissue patterns of each specimen. The detailed osteohistological descriptions of each element can be found in Data S1 and Figures S1–, S10.

**FIGURE 1:**
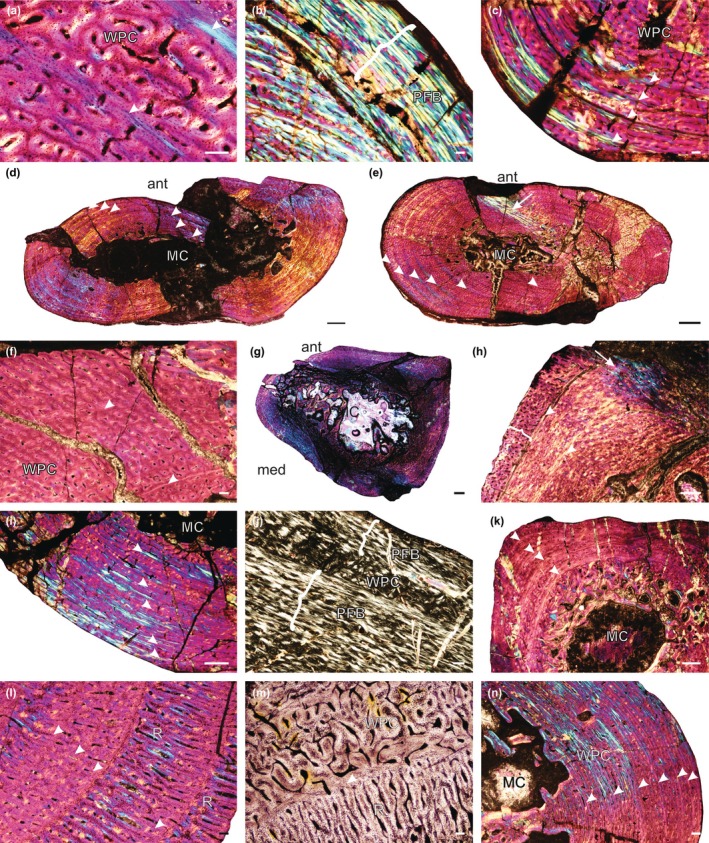
Osteohistology of various gorgonopsians. (a) *Cyonosaurus* sp. ulna SAM‐PK‐K10428b showing a highly vascularized WPC and two annuli; (b) SAM‐PK‐10188a humerus showing a peripheral region (brackets) of slower growing PFB; (c) a rapidly forming WPC interrupted by four growth marks; (d) SAM‐PK‐10188b overview of the femur showing six growth marks; (e) SAM‐PK‐10188e overview of the tibia showing a WPC interrupted by six growth marks; (f) SAM‐PK‐K10000d tibia showing indistinct growth marks; (g) overview of humerus *Gorgonops torvus* SAM‐PK‐K4460; (h) close‐up of the humerus showing a peripheral growth spurt (brackets) and Sharpey's fibers (arrow); (i) *Aelurognathus tigriceps* SAM‐PK‐K10035a humerus showing five growth marks; (j) SAM‐PK‐K10110 humerus showing alternating regions of a WPC and PFB (brackets); (k) SAM‐PK‐K8622c fibula showing a WPC and four growth marks; (l) SAM‐PK‐K8623d femur showing five annuli; (m) SAM‐PK‐K10622d femur showing a WPC and a region of radiating vascular canals; (n) CGS 391‐83d femur showing a WPC. Arrowheads indicate growth marks. Ant, anterior surface; MC, medullary cavity; med, medial surface; PFB, parallel‐fibered bone; R, radial canals; WPC, woven‐parallel complex. Scale bars b–e, g, n = 1000 μm; a, f, h–k = 500 μm; l, m = 100 μm.

Two specimens were identified as *Cyonosaurus* sp., a small gorgonopsian with a maximum skull length of 20 cm (Benoit et al., 2024). They include SAM‐PK‐K10428, a well‐preserved ulna, which contains a highly vascularized woven‐parallel bone tissue complex (WPC) (de Buffrénil & Quilhac, 2021; Prondvai et al., 2014) interrupted by two annuli, indicating a temporary decrease in growth rate (Figure 1a; Figure S1a,b). The second specimen, SAM‐PK‐10188 (Figure 1b–e; Figure S1c–r) includes several limb bones (humerus, femur, radius, ulna, tibia, rib) re‐identified from *Scylacops* to *Cyonosaurus* and also displays a similar woven‐parallel bone structure. The elements of SAM‐PK‐10188 exhibit five annuli, with narrower zones toward the periphery, suggesting that it was a subadult at death. The humerus exhibits a larger amount of parallel‐fibered bone toward the periphery in some places (Figure 1b), but growth continued rapidly in other regions (Figure 1c). No outer circumferential lamellae (OCL), essentially indicating a cessation in growth, were observed, however, indicating that maximum size had not been attained (Figure 1d,e).

The osteohistology of SAM‐PK‐K10000, previously classified as *Aelurognathus* but currently unconfirmable, has been further examined in new sections of the humerus, radius, ulna, and tibia. The examined bones display a highly vascularized WPC interrupted by faint annuli (Figure 1f; Figure S2). Three annuli were identified in the humerus and radius, and two in the ulna and tibia, with vascular canal orientations mostly longitudinal. Given that a maximum of three annual growth marks were observed in SAM‐PK‐K10000 and it is larger than the *Cyonosaurus* specimens SAM‐PK‐K10428 and SAM‐PK‐10188, it likely does not represent a specimen of *Cyonosaurus*. The humerus of SAM‐PK‐10188 is also larger than SAM‐PK‐K10035, which has been identified as *Aelurognathus* (described below), but is ontogenetically younger by a large margin, suggesting that SAM‐PK‐K10000 does not belong to *Aelurognathus* (see Data S1 for a detailed discussion).

The humerus of SAM‐PK‐K4460 has been identified as *Gorgonops torvus* (Figure 1g; Figure S3a–c), a medium‐sized gorgonopsian with a maximum skull length of 35 cm (Kammerer, 2016). The cortex features a highly vascularized WPC, with vascular canal orientations that are a mix of reticular and longitudinally oriented primary osteons. Two growth marks are present: a faint inner annulus and an outer annulus, the latter of which transitions into a line of arrested growth (LAG) in some areas. Following the second growth mark, a growth spurt is noted, characterized by larger, more reticular canals in the outer region (Figure 1h). This rapid growth indicates that the specimen was rapidly growing and was ontogenetically young at the time of death.

The humerus and ulna of SAM‐PK‐K10035 have been identified as *Aelurognathus tigriceps*, a species with a basal skull length of 30 cm (Kammerer, 2016), comparable to *Gorgonops*. The bone tissue of the humerus resembles that of SAM‐PK‐K4460, but it features a more laminar vascular canal orientation and shows five growth marks of parallel‐fibered bone (Figure 1i; Figure S3d–f), indicating that this specimen was ontogenetically older despite a similar size. The ulna shows vascular canals within a WPC that range from longitudinally oriented primary osteons to simple, reticular, and short radiating canals (Figure S3i). Both bones were still actively growing at the time of death.

SAM‐PK‐K10110 has not yet been assigned to a species but may represent a new taxon, as suggested by C. Kammerer (pers. comm., 2023). The specimen includes skull material, which may allow for a future detailed description. Its primary cortex features a WPC, with parallel‐fibered bone becoming more prominent toward the sub‐periosteal surface. Three distinct growth marks are visible, with a possible fourth partially remodeled mark in the inner cortex; in some regions, these growth marks form LAGs or double LAGs (identified as two closely spaced LAGs). Other areas exhibit annuli of lamellar bone that var from narrow to unusually wide bands (Figure 1j; Figure S3j–l), with the outermost band reaching the sub‐periosteal surface, indicating a decrease in overall growth rate. Although in certain locations, rapid growth continued after the outer LAG, there is an increase in the slower‐forming parallel‐fibered bone toward the periphery, indicating a subadult status and possibly the onset of reproductive maturity (defined by de Buffrénil & Quilhac, 2021 as a decrease in growth rate, i.e., increased prevalence of slower‐forming bone tissues, an overall decrease in zone width, or the presence of an OCL).

SAM‐PK‐K8622 includes a femur, tibia, and fibula, all exhibiting a WPC interrupted by narrow annuli and/or LAGs, with four growth marks evident in each limb bone (Figure 1k; Figure S4a–i). The presence of four growth marks indicates that this individual was a subadult, but there is no evidence of a decrease in growth rate, and the ulna reveals a small area of highly vascularized bone tissue at the periphery in places, indicating active growth at death. SAM‐PK‐K8623, found near SAM‐PK‐K8622 on the same farm, has not yet been assigned a species but may represent the same taxon. All limb bones exhibit a highly vascularized WPC, with up to six growth marks appearing as faint annuli that are difficult to observe in normal light. Vascular orientation is primarily reticular, with more radial canals noted in the radius, and clear radial vascular canals in the ulna indicating rapid growth spurts (Figure 1l; Figure S5a–l). There is no indication of decreasing growth rate toward the periphery, suggesting the individual was still actively growing at death, despite being at least 6 years old.

Two femora from specimen SAM‐PK‐K10622 were thin sectioned, but due to their incompleteness, sections could not be taken from the midshaft. Despite this limitation, the specimen displays alternating zones with differing vascular orientations. Faint annuli are visible under cross‐polarized light, bounding zones that contain bone tissues with radiating vascular canals, again indicating bursts of rapid growth as WPC with vascular canals primarily arranged as longitudinally oriented primary osteons, transitioning to a reticular pattern in some areas.

A humerus, radius, ulna, and femur were sectioned from specimen CGS AF 391–83, which has not been identified to genus level. All elements exhibit a highly vascularized WPC, with vascularization decreasing toward the sub‐periosteal surface in the humerus, radius, and femur (Figure 1n; Figure S6d–o). The ulna features a wide annulus of parallel‐fibered bone at its surface, making it unclear if vascularization had decreaseds in this element. There are multiple growth marks, and with a gradual decrease in vascularization, and increasing parallel‐fibered bone, these features suggest an overall reduction in growth rate, suggesting that CGS AF 391–83 was a subadult at the time of death and may have reached reproductive maturity.

**FIGURE 2:**
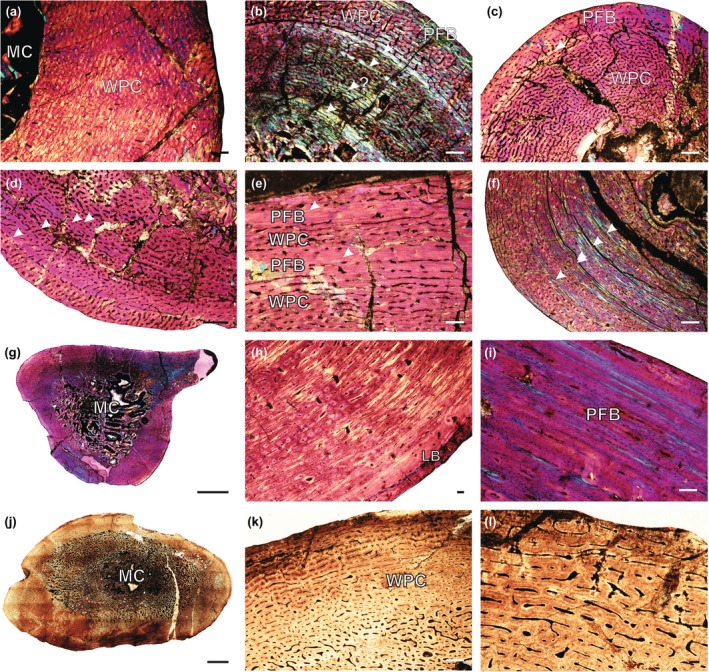
Osteohistology of various gorgonopsians. (a) CGS FL‐43d femur showing an uninterrupted WPC; (b) BP/1/4940 humerus showing four annuli and a peripheral region of PFB; (c) SAM‐PK‐K6415 radius showing an annulus and possibly a second indicated by the PFB; (d) SAM‐PK‐K6415 ulna showing three LAGs (with a double LAG) and rapidly forming bone at the periphery; (e) SAM‐PK‐K6407 femur showing two wide regions of PFB; (f) BP/1/4259 humerus showing four LAGs in a WPC; (g) overview of humerus *Arctops willistoni* BP/1/1533; (h) the humerus showing an outer region of lamellar bone; (i) close‐up showing a wide outer region of PFB; (j) overview of *Inostrancevia africana* NMQR 4000 femur; (k) femur showing WPC; (l) close‐up of the femur showing a WPC. Arrowheads indicate growth marks. LB, lamellar bone; MC, medullary cavity; PFB, parallel‐fibered bone; WPC, woven‐parallel complex. Scale bars g, j = 5000 μm; b–f, k, l = 500 μm; a, h, i = 100 μm.

CGS FL‐43 is an articulated skeleton of an indeterminate gorgonopsian from which the humerus, femur, both radii, ulnae, and tibiae were sectioned. The predominant tissue type is a WPC, with vascular arrangements varying from mostly laminar in the humerus and femur (Figure 2a; Figure S7a–r) to a mix of longitudinal primary osteons and reticular canals in the radii and ulnae. A mid‐cortical LAG is present in the ulnae, and growth marks were observed in the radii, left tibia, and humerus, indicating the individual was likely in its second year of growth and may represent a late juvenile or early subadult. The vascularized WPC reaches the sub‐periosteal surface and suggests a relatively young ontogenetic stage, possibly indicating this individual is a juvenile of a larger species.

The humerus of BP/1/4940 is an indeterminate gorgonopsian. The bone tissue displays a highly vascularized WPC, with vascular canals forming plexiform and reticular networks that extend to the sub‐periosteal surface. Four annuli of parallel‐fibered bone traverse the cortex, and a broad region of parallel‐fibered bone is observed in patches around the sub‐periosteal surface, which may indicate an overall decrease in growth rate (Figure 2b; Figure S8a–d). This is difficult to confirm, however, as other regions at the periphery show rapidly forming WPC. This suggests the very beginning of a decreased growth rate, and thus it is possible that reproductive maturity had been reached.

Specimen SAM‐PK‐K6415, initially identified as *Arctognathus*, has been reclassified as *Tigricephalus kingwilli*, although there is ongoing debate about its relationship with *Aelurognathus* (Kammerer, 2016; C. Kammerer, pers. comm., 2023, see Data S1 for a detailed discussion). Further cranial studies are needed to clarify this classification. The radius and ulna display a highly vascularized WPC with vascular patterns being mostly reticular. The radius shows a clear LAG and possible second growth mark (Figure 2c; Figure S8e–g), whereas the ulna exhibits three LAGs (the first being a double LAG) but remains highly vascularized (Figure 2d; Figure S8h–j), indicating that the individual had likely not reached reproductive maturity. Overall, the limb bones of SAM‐PK‐K6415 are larger than those of the *Aelurognathus* specimen SAM‐PK‐K10035, suggesting that it is ontogenetically younger and may represent a distinct species.

Specimen SAM‐PK‐K6407 is an indeterminate gorgonopsian from which a humerus and femur were thin sectioned. Both elements exhibit a highly vascularized WPC with five growth marks in the humerus, and four in the femur. The outer two LAGs in the femur are associated with wide regions of parallel‐fibered bone (Figure 2e; Figure S9a–f). This specimen likely represents a subadult, but there is no evidence to suggest that reproductive maturity had been reached.

Specimen BP/1/4259, initially referred to *Cyonosaurus* but re‐identified as an indeterminate gorgonopsian by Kammerer (pers. comm., 2023), includes a humerus, radius, and ulna. All three bones feature a well‐vascularized WPC as their primary cortex, with varying vascular arrangements. Four LAGs were noted in the humerus (Figure 2f; Figure S9g–o) and ulna, and three in the radius, with no decrease in zone width toward the peripheries, indicating that the animal was still rapidly growing at death. Compared to positively identified *Cyonosaurus* specimens, BP/1/4259 is larger and likely represents a different, large‐bodied gorgonopsian species.

Specimen BP/1/1258 (Figure S10a–i) is identified as a large‐bodied indeterminate gorgonopsian, featuring a humerus, radius, and partially preserved ulna. The humerus has a highly vascularized WPC with a predominantly laminar vascular orientation, whereas the radius and ulna also show well‐vascularized tissues, with the radius exhibiting a slight radial orientation in areas containing Sharpey's fibers (representing areas of muscle insertion). No growth marks were observed, indicating that this relatively large specimen was still growing rapidly at the time of death, and may have been a young individual of a large‐bodied species.

Specimen BP/1/1533 (Figure 2g; Figure S10j–m) has been confirmed as *Arctops willistoni*, a medium‐sized gorgonopsian with a maximum basal skull length of 27 cm (Kammerer, 2016). The compact cortex primarily consists of a highly vascularized WPC with mostly laminar vascular canals. Two relatively closely spaced annuli are present toward the outer cortex, with the zone between them showing more parallel‐fibered bone in some areas. The outermost annulus is notably wide, indicating a possible decrease in growth rate at the time of death (Figure 2h). The presence of more slowly formed parallel‐fibered bone suggests that the individual may have reached reproductive maturity before its death (Figure 2i).

NMQR 4000 (Figure 2j; Figure S10n–p) is identified as a new species of South African gorgonopsian, named *Inostrancevia africana*. This species is represented by two skulls and a skeleton, with the largest skull measuring 44 cm in length (Kammerer et al., 2023). The bone tissue consists of a highly vascularized WPC (Figure 2k,l), with various vascular canal orientations. Overall, the high vascularization and lack of slower‐forming bone tissues indicate that the individual was still ontogenetically young at the time of death, having likely experienced approximately 10 mm of growth in a single year. Despite its size, it had not reached reproductive maturity and would have continued to grow significantly had it lived to adulthood.

### 3.2 Cortical thickness

**FIGURE 3:**
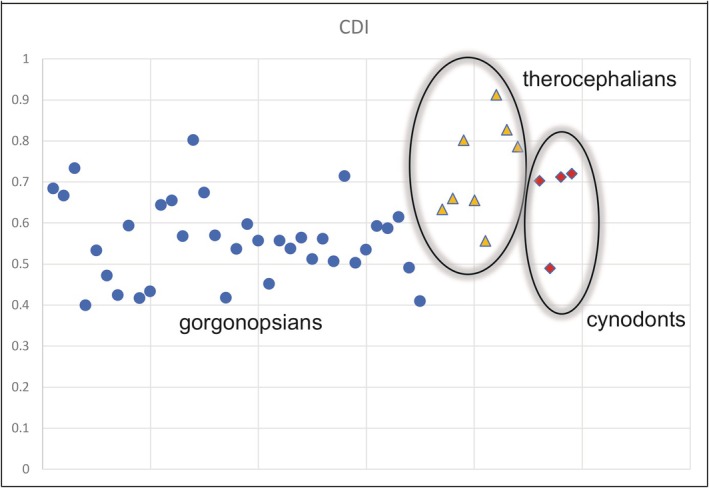
CDI (i.e., bone wall thickness) for gorgonopsians (blue circles), therocephalians (yellow triangles), and non‐mammaliaform cynodonts (red diamonds) used in this study.

A series of values were collected to quantify the cortical thickness of the limb bones and the transition zone from open medullary cavity to compact cortex (Data S2). The results show that the cortical thickness (CDI) falls within the range of other theriodonts (0.557 to 0.913 see Figure 3; Table S2). Most of the bones contain open medullary cavities except for several humeri. Of note is SAM‐PK‐K8623 where the medullary cavities of the humerus, radius, and ulna are completely infilled with bony trabeculae (Figure S5), leaving only very small clear cavities in the radius and ulna. The femoral cortex is slightly thinner, but its medullary cavity is also partially filled. The cortical thickness of the bones in this specimen is higher than most of the other bones in the sample. Although SAM‐PK‐K8623 has not been positively identified to genus level, it is worth noting this difference in bone microanatomy, which might reflect ecological differences from other gorgonopsians. The femur of *Inostrancevia africana* (NMQR 4000) reveals distinct bone microanatomy, including a large medullary cavity almost completely infilled with fine bony trabeculae, resulting in a relatively thin compact cortex (Figure 2j). This differs from any of other femora in the sample. The animal's large body size may have influenced its bone microanatomy, resulting in a reduction in cortical thickness and overall mass. However, it maintained structural strength by filling the medullary cavity with bony struts.

## 4 DISCUSSION

### 4.1 Gorgonopsian growth and ontogeny

The elements of all the gorgonopsian specimens reveal rapid growth in the form of a highly vascularized woven‐parallel complex. Vascular arrangements vary between elements, but larger bones tend to have a laminar arrangement, and the radius and ulna often contain more longitudinally oriented primary osteons compared to other elements. A few elements contain zones with radial vascular canals, indicating short bursts of even faster bone deposition (Margerie, de, et al., 2004). This is best presented in SAM‐PK‐K8623 where almost every zone is dominated by these radial canals, indicating extremely rapid growth during the favorable seasons. The bone tissue is interrupted, in most specimens, by annual growth marks.

In most cases, the individuals in this study were subadults when they died (72% of the sample), apart from CGS FL‐43, BP/1/1258, and NMQR 4000 which may represent juveniles or at most early subadults. NMQR 4000, identified as *Inostrancevia africana* (with a basal skull length of 44 cm), is a remarkably large individual for it to be a juvenile and probably represents an early subadult. Even if one or two growth marks have been destroyed, the minimum of 10 mm of bone deposition in one season (Data S2, Table S1) indicates the fastest growth compared to any of the other gorgonopsian specimens in this study, and it clearly had not reached subadult status.

**FIGURE 4:**
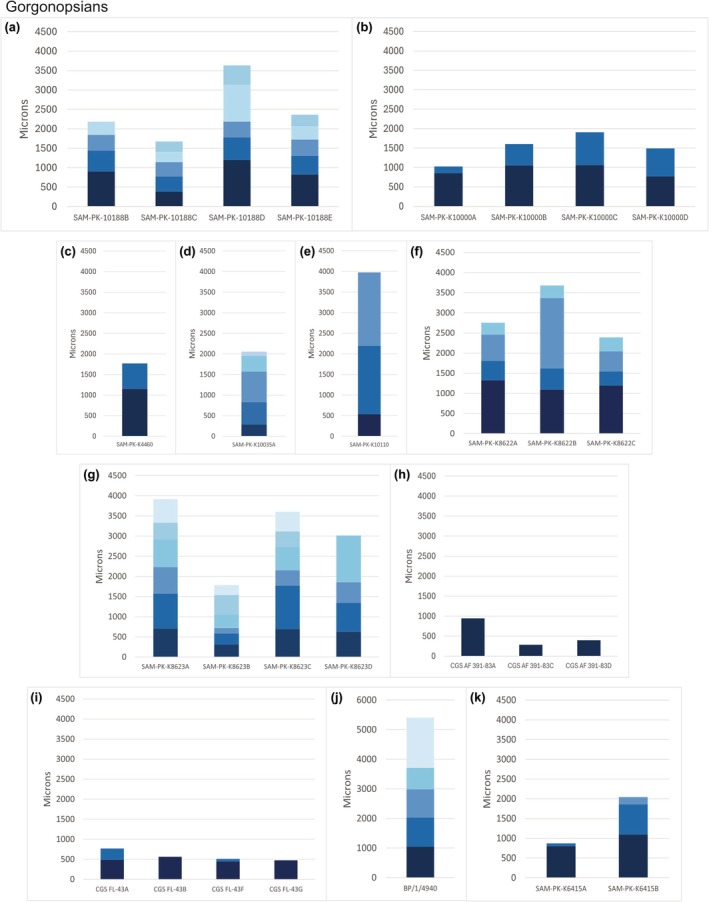
Zone widths in microns for the gorgonopsian elements that could be measured and compared against similar‐sized therocephalians and non‐mammaliaform cynodonts. Growth mark one is the *x*‐axis of each graph; each subsequent zone is a different blue. The darkest blue is between growth marks one and two (Data S2 for raw data). (a) SAM‐PK‐K10188 femur, radius, ulna, tibia; (b) SAM‐PK‐K10000 humerus, radius, ulna, tibia; (c) SAM‐PK‐K4460 humerus; (d) SAM‐PK‐K10035 humerus; (e) SAM‐PK‐K10110 humerus; (f) SAM‐PK‐K8622 femur, tibia, fibula; (g) SAM‐PK‐K8623 humerus, ulna, radius, femur; (h) CGS AF 391–83 humerus, ulna, femur; (i) CGS FL‐43 humerus, right radius, left ulna, left radius; (j) BP/1/4940 humerus; (k) SAM‐PK‐K6415 radius, ulna.

Zone widths are variable within individuals in four specimens (SAM‐PK‐10188, SAM‐PK‐K8622, SAM‐PK‐K8623, BP/1/4940), with some of the outer zones being thicker than the innermost zones. This suggests that gorgonopsians were relatively dependent on environmental changes. Based on the amount of bone deposition between the first and second growth marks, the specimens can be loosely divided into three categories: those where the bone deposition is equal to or less than 500 μm, those where this zone is approximately 1000 μm thick, and those where this zone is more than 2000 μm thick (Data S2; Figure 4). The larger specimens tend to be closer to the 1000 μm mark (Figure 4). Three specimens, BP/1/1258, BP/1/1533, and NMQR 4000, show a maximum deposition of 7000 μm, 2000 μm, and 10,000 μm, respectively, measured from the medullary cavity to the bone periphery. BP/1/1533 has been identified as *Arctops willistoni* and NMQR 4000 as *Inostrancevia africana*. *Inostrancevia africana* is a large‐bodied taxon with a basal skull length of 44 cm. *Arctops* is considered a mid‐sized gorgonopsian with a basal skull length of 27 cm (Kammerer, 2016), which is smaller than other gorgonopsians such as *Phorys*, *Gorgonops*, *Lycaenops*, *Smilesaurus*, and *Aelurognathus*, which have basal skull lengths of 30 cm or more (Kammerer, 2016). BP/1/1533 deposited 2000 μm of bone in this inner zone that was measured (Figure 4), which is most similar to SAM‐PK‐K10110, but otherwise differs from the other specimens. BP/1/1258 was recovered from the *Cistecephalus* Assemblage Zone, and it represents a large taxon. It likely represents a taxon belonging to the Rubidgeini, which are all large animals with a basal skull length of more than 36 cm (Kammerer, 2016) and include *Rubidgea atrox*, *Clelandina rubidgei*, and *Dinogorgon rubidgei* or to the non‐rubidgeine *Leontosaurus vanderhorsti*, which has a maximum basal skull length of approximately 40 cm (Kammerer, 2016).

SAM‐PK‐K10000 was still rapidly growing and likely an early subadult when it died, and the specimen already had a basal skull length of 18 cm. Thus, it is unlikely that SAM‐PK‐K10000 represents one of the smaller gorgonopsian species such as *Cyonosaurus*, *Aelurosaurus*, *Eriphostoma*, *Cynariops*, or *Arctognathus*. This specimen was recovered from the *Endothiodon* Assemblage Zone, upper *Tropidostoma*‐*Gorgonops* Subzone where several gorgonopsian taxa occur, namely *Gorgonops*, *Aelurosaurus*, *Lycaenops*, *Cynariops*, *Aelurognathus*, *Smilesaurus*, and *Arctops* (Day & Smith, 2020). *Aelurognathus* has been ruled out as a possibility (C. Kammerer, pers. Comm., 2023), and thus SAM‐PK‐K10000 might represent *Gorgonops*, *Lycaenops*, *Smilesaurus*, or *Arctops*.

SAM‐PK‐K10110 may represent a new species of gorgonopsian (C. Kammerer, pers. Comm., 2023). There are no unique osteohistological characteristics in the humerus of this specimen, however. The WPC is interrupted by three faint annuli similar to other taxa. Thus, unfortunately the bone microstructure cannot provide any distinguishing features about this taxon. The osteohistology does differ slightly from a similar‐sized indeterminate gorgonopsian, SAM‐PK‐K4460, in that the latter humerus expresses a burst of rapid growth after the second growth mark. Otherwise, both bones are similar in being 2–3 years old and not showing any evidence of decreased growth rates.

SAM‐PK‐K8622 and SAM‐PK‐K8623 were found near each other on the same farm Rust 126, Doornplaats, Graaff‐Reinet in the *Daptocephalus* Assemblage Zone. They are similar in size and ontogenetic age, with SAM‐PK‐K8623 being slightly larger and older by 1 or 2 years. Both were still growing rapidly at the time of death. The elements from SAM‐PK‐K8622 belong to the hind limb and those of SAM‐PK‐K8623 belong to the forelimb, which makes comparisons difficult. However, a femur was sectioned from both specimens, and the bone tissue patterns are similar, with nothing to suggest that they belong to different species. It is noteworthy that the ulna of SAM‐PK‐K8623 exhibits four cycles of radiating canals, indicative of very rapid growth. The amount of bone deposition between growth marks is less than that of the larger specimens, but this is likely related to body size and not a difference in absolute growth rates. CGS AF 391–83 is similar in size and ontogenetic age to SAM‐PK‐K8623. Similar amounts of bone tissue were deposited between growth marks. There does appear to be a slight decrease in growth rate toward the bone periphery of the humerus, ulna, and femur, suggesting the onset of slower growth. CGS FL‐43 on the other hand is similar in size to CGS AF 391–83 but was clearly ontogenetically younger, and thus these two specimens are unlikely to represent the same species. Unfortunately, the localities of CGS AF 391–83 and CGS FL‐43 are unknown, making it impossible to assign these specimens to possible species.

### 4.2 Cortical thickness

The cortical thickness of the gorgonopsian limb bones was quantified and compared with similar‐sized therocephalians (scylacosaurid, *Lycosuchus*, *Moschorhinus*) and non‐mammaliaform cynodonts (*Cynognathus*, *Diademodon*). The cortical thickness of gorgonopsians was comparable to that of other theriodonts. In fact, the therocephalian *Moschorhinus* has the highest cortical thickness among this group (Figure 3; Data S2).

### 4.3 Growth rates

**FIGURE 5:**
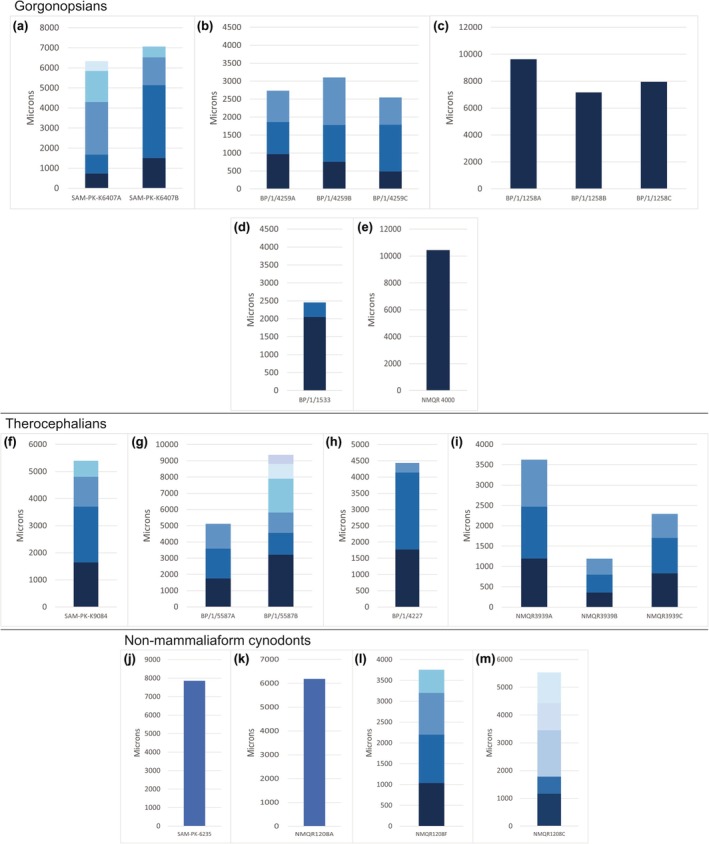
Zone widths in microns for the gorgonopsian elements that could be measured and compared against similar‐sized therocephalians and non‐mammaliaform cynodonts. Growth mark one is the *x*‐axis of each graph; each subsequent zone is a different blue. The darkest blue is between growth marks one and two (Data S2 for raw data). Gorgonopsians, (a) SAM‐PK‐K6407 humerus, femur; (b) BP/1/4259 humerus, radius, ulna; (c) BP/1/1258 humerus, radius, ulna; (d) BP/1/1533 humerus; (e) NMQR 4000 femur; Therocephalians; (f) *Lycosuchus vanderrieti* SAM‐PK‐K9084 ulna; (g) scylacosaurid BP/1/5587 humerus, ulna; (h) *Moschorhinus kitchingi* BP/1/4227 humerus; (i) *Moschorhinus kitchingi* NMQR 3939 humerus, radius, tibia; Non‐mammaliaform cynodonts, (j) *Cynognathus crateronotus* SAM‐PK‐6235 femur; (k) *Cynognathus crateronotus* NMQR 1208a humerus; (l) *Diademodon tetragonius* NMQR 1208f femur; (m) *Diademodon tetragonius* NMQR 1208c humerus.

Zone widths of the gorgonopsians were compared to those of other similar‐sized eutheriodonts (therocephalians and non‐mammaliaform cynodonts). It should be noted that this is merely a rough comparison as ontogenetic status varies between the specimens, and thus a detailed comparison cannot be done here. However, it can be noted that the eutheriodonts generally deposit 1000 μm of bone tissue between the first and second growth marks, which differs from five of the gorgonopsian specimens (Figures 4, 5). Larger specimens (including *Arctops* and *Inostrancevia africana*) deposited larger amounts of bone tissue to the eutheriodonts at this stage of life. Within individual variation was distinct in the gorgonopsians, SAM‐PK‐10188, CGS 391–83, SAM‐PK‐K6415, and SAM‐PK‐K6407, where values ranged between 500 μm and 1000 μm.

### 4.4 Reproductive maturity

Evidence from the bone tissues indicate that some gorgonopsians may have reached reproductive maturity at larger sizes compared to Early Triassic therapsids. This stage is usually identified by decreased zone widths toward the sub‐periosteal surface and/or an increase in slower‐forming bone tissues such as parallel‐fibered or lamellar bone (e.g., Castanet et al., 2004; Erickson et al., 2007; Hutton, 1986; Kohler et al., 2012; Lee & Werning, 2008; Straehl et al., 2013). Only 22% of the specimens in this study show any evidence for the onset of reproductive maturity. This observation suggests that gorgonopsians could have developed a growth strategy favoring larger body sizes before reproduction (i.e., delayed reproductive maturity).

### 4.5 Gorgonopsians and the Permo‐Triassic mass extinction

The abundance of growth marks in most specimens indicates a dominance of subadults in the sample. This differs from what has been observed in the Early Triassic, where mostly juveniles have been recovered (Botha‐Brink et al., 2016). It has been suggested that therapsids died at young ages in the Early Triassic following the Permo‐Triassic mass extinction due to the unpredictable environment that ensued after the event (Botha‐Brink et al., 2016). The gorgonopsians in this study are all Permian but range in time as specimens were sampled from the *Endothiodon* Assemblage Zone (and possibly lower given that SAM‐PK‐K4460 may come from the underlying *Tapinocephalus* Assemblage Zone) through to the top of the *Daptocephalus* Assemblage Zone, which contains the beginning of the Permo‐Triassic mass extinction. This suggests that during the Permian, gorgonopsians were able to reach later ontogenetic stages than Early Triassic taxa, similar to what has been reported for other Permian therapsids (Botha‐Brink et al., 2016). This supports observations that there were better (i.e., more predictable, less arid) environmental conditions in the Permian allowing species to live longer than those in the Early Triassic (Botha‐Brink et al., 2016; Rey et al., 2016; Smith & Botha‐Brink, 2014; Viglietti et al., 2013).

## 5 CONCLUSION

The osteohistological analysis of gorgonopsian specimens reveals a pattern of rapid growth marked by highly vascularized woven‐parallel bone tissue. The presence of numerous growth marks, alongside evidence of variable zone widths, indicates a growth history that may suggest longer lifespans and slower growth rates compared to therapsids from the Early Triassic. The high vascularity and the observed growth patterns imply that gorgonopsians could grow quickly during favorable seasons, yet the overall presence of multiple growth marks suggests they had the potential to live longer and mature slower than their Early Triassic counterparts. The findings indicate that gorgonopsians thrived in a more stable environment during the Permian, as evidenced by the dominance of subadult specimens in the sample. The ability of gorgonopsians to reach later ontogenetic stages aligns with hypotheses that more favorable environmental conditions facilitated larger body sizes. The growth patterns observed—particularly the significant deposition of bone during optimal periods—suggest that these conditions were conducive to sustained growth and development, allowing gorgonopsians to achieve larger sizes than those typically seen in Early Triassic therapsids. Instead, Early Triassic therapsids were either juveniles or reached reproductive maturity within a year, likely reflecting harsher conditions that led to higher mortality rates at younger ages. The presence of numerous growth marks in the gorgonopsian specimens suggests that they had more opportunities to mature before facing environmental challenges, thus reinforcing the notion of a stable habitat conducive to growth. However, delayed reproductive maturity, possibly due to large body size, does not appear to have been advantageous during the PTME as most Early Triassic taxa moved the onset of reproductive maturity forward (Botha‐Brink et al., 2016). This growth strategy may have played a role in the demise of the gorgonopsians during the PTME and should be investigated further.

## AUTHOR CONTRIBUTIONS

JB conceptualized this study, and investigated and interpreted the osteohistology of the specimens.

## CONFLICT OF INTEREST STATEMENT

I declare that I have no conflict of interest.

## Supporting information


Figure S1.

Figure S2.

Figure S3.

Figure S4.

Figure S5.

Figure S6.

Figure S7.

Figure S8.

Figure S9.

Figure S10.

Data S1.

Data S2.

## Data Availability

The data that support the findings of this study are openly available in Morphosource under project ID 000668546.
