# Supplementary material for: The osteohistology of gorgonopsian therapsids and implications for Permo‐Triassic theriodont growth
Source: J Anat. 2024 Dec 20;246(6):987–1000. doi: 10.1111/joa.14201 (PMC12079768; doi:10.1111/joa.14201)
Supplement: Supplementary file 11 — Data S1. [file JOA-246-987-s002.pdf]

Supplementary Information 1

Detailed Osteohistological Description

As several specimens have not been identified to generic level the following osteohistological descriptions are organized based on the circumference of the elements for each specimen, from smallest to largest. Generally, the humerus and femur were used to order the specimens, but there were some cases where these elements were not cut at the middle of the midshaft and thus, the circumferences were overestimated. In these cases, both the radius and ulna from a particular specimen were used to place the specimens in order. However, given that the specimens in this sample represent different species, this organization is not essential for any analyses.

SAM-PK-K10428 and SAM-PK-10188 (Figure S1)

Both specimens have been positively identified as *Cyonosaurus*. This genus comprises several species and the specimens cannot be identified further, thus these individuals are referred to as *Cyonosaurus* sp. SAM-PK-K10428 is an ulna that was sectioned slightly off the middle of the midshaft, thus, the circumference was not measured as it would have been overestimated and no binary was made. However, it is important to include this specimen as it is small, and the bone tissue is well preserved. The bone tissue comprises a woven-parallel complex (Prondvai et al., 2014; Buffrénil et al., 2021), which is defined as a mixture of woven and parallel-fibered bone. The rapidly growing woven bone is identified as such due to the presence of large, globular, irregularly spaced osteocyte lacunae (indicating static osteogenesis, Prondvai et al., 2014). The slower growing parallel-fibered bone is present within the primary osteons, where the osteocyte lacunae are more organized (indicating dynamic osteogenesis, Prondvai et al., 2014). In this bone they are numerous and primarily longitudinally oriented with short anastomoses. Botha-Brink et al. (2016) quantified the mid-cortical vascularization as 7.5%. Despite the small size of this element, two annuli consisting of slower growing parallel-fibered bone and indicating annual decreases in growth rate, were observed. There is no indication of decreased growth in the form of decreased vascularization or a transition to slower forming bone tissue at the bone periphery. Moreover, no outer circumferential lamellae (OCL, also known as an External Fundamental System), indicating that growth had essentially ceased, was observed. A region of compacted coarse cancellous bone (CCCB) is present in this section and the compact cortex is not particularly thick, further indicating that the section was not taken from the middle of the midshaft.

The humerus, femur, radius, ulna, tibia and a rib were sectioned from SAM-PK-10188 (previously identified as *Scylacops* in Ray et al., 2004 and Ray and Chinsamy, 2012, which is now considered a junior synonym of *Aelurognathus*, but has been reidentified as *Cyonosaurus* by C. Kammerer, 2023). The humerus has been severely crushed so the widths of the zones between growth marks were not measured. The femur is slightly crushed, but the compact cortex is preserved well enough to measure the width of the zones between growth marks. The rest of the elements are intact. The specimens exhibit similar bone tissues to SAM-PK-K10428, with a highly vascularized woven-parallel complex dominating the cortex. Vascular canal organization ranges from plexiform in the humerus and reticular in the rest of the limb bones, with the rib exhibiting primarily longitudinally oriented primary osteons and simple canals. Ray et al. (2004) quantified the mid-cortical vascularization for the ulna (3.8%), radius (2.5), femur (4.3%), fibula (3.6%) and rib (2.2%). However, Botha-Brink et al. (2016) quantified the vascularization for the femur, fibula and rib and recovered a higher vascularization at 7.8%, 7.5% and 3.5%, respectively. These numbers are similar to the SAM-PK-K10428 ulna. Five annuli were observed in each element (similar to that noted in Ray et al., 2004). Ray et al. (2004) also noted that only the femur contained a laminar vascular arrangement. There are small patches of laminar canals in the femur, but there are more reticular canals. Regardless, these arrangements are indicative of rapidly forming bone tissues. The new sections in this study also revealed plexiform bone in the humerus in contrast to that reported in Ray et al. (2004). The annuli are comprised of parallel-fibered bone but are often difficult to see clearly in normal light. This is the case for many of the elements in the study sample, where polarized light or polarized light with a lambda compensator had to be used to observe the growth marks. Despite the small size of this individual, a decrease in growth rate (i.e. zones become narrower towards the bone periphery) was observed in the humerus, femur, radius and tibia of SAM-PK-10188. This indicates that SAM-PK-10188 was likely a subadult at the time of death and would not have grown considerably larger (Ray et al., 2004 noted a basal skull length of 18 cm, close to the maximum known size). However, an OCL was not observed in any element. Ray and Chinsamy (2012) reported a region of lamellar bone at the sub-periosteal surface of the humerus. In this study, the new sections show similar peripheral tissues, but this feature is regional as no OCL was observed in the rest of the humerus. The decrease in growth rate supports the proposed identification of this specimen as *Cyonosaurus*. Peri-medullary trabeculae are present in each element, but they do not fill the medullary cavity completely. Secondary remodeling in the form of resorption cavities and secondary osteons are limited to

the inner cortex. Some of the secondary osteons in the radius overlap slightly, but they do not form dense Haversian bone as described by Ray and Chinsamy (2012). Sharpey's fibers, indicating areas of muscle insertion are seen in the radius (on the posteromedial side) and tibia (on the anterior side) where they extend through to the inner cortex in the latter bone.

SAM-PK-K10000 (Figure S2)

The osteohistology of SAM-PK-K10000 (previously identified as *Aelurognathus*, but the generic identification cannot be confirmed at present) was briefly described in Ray et al. (2004) and Ray and Chinsamy (2012). New sections of the humerus, radius, ulna and tibia were made for this study. The femur that was published in these earlier studies is not included here as the sections were made too far from the midshaft to provide any relevant information. The humerus, radius, ulna and tibia exhibit similar tissues to *Cyonosaurus*, i.e. a highly vascularized woven-parallel complex interrupted by faint annuli. The growth marks are very difficult to see and are sometimes only visible in a particular light. Three annuli were observed in the radius and two in the humerus, ulna and tibia. The vascular canal orientation is mostly longitudinally oriented primary osteons with some short anastomoses. Ray et al. (2004) reported a vascularization of 4.7% and Botha-Brink et al. (2016) reported a similar amount of 5.5%. There is no clear decrease in vascularization towards the bone periphery, nor is there a clear indication of narrowing zones towards the sub-periosteal surface. At least three growth marks are present in this individual, but it is larger than SAM-PK-K10428 and SAM-PK-10188, suggesting that SAM-PK-K10000 cannot be referred to *Cyonosaurus*. The humerus has a larger circumference than the humerus of specimen SAM-PK-K10035, which has been identified as *Aelurognathus*, and yet the SAM-PK-K10000 individual contains up to three annuli and SAM-PK-K10035 contains five annuli. This also suggests that SAM-PK-K10000 does not belong to *Aelurognathus* (see below for description of SAM-PK-K10035). The cortices are not particularly thick, but many of the elements have broken trabeculae within the medullary cavity. Given that they are broken fragments it is unlikely that the medullary cavities were completely infilled and were probably similar to the bones described above for *Cyonosaurus*. There are broken fragments of an inner lining of endosteal bone in the humerus, radius and ulna. The extensive compacted coarse cancellous bone noted in Ray et al. (2004) is not observed here, probably because these sections were taken closer to the midshaft than those in Ray et al. (2004). There is some secondary remodeling in the form of a few resorption cavities in the innermost cortex, but it is not extensive. Two large regions of Sharpey's fibers were observed in the anterior and posterior sides of the tibia.

SAM-PK-K4460 (Figure S3)

The humerus of SAM-PK-K4460 was sectioned and the specimen has been identified as *Gorgonops torvus*. The cortex comprises a highly vascularized woven-parallel complex similar to the other specimens. The vascular canal orientation is a mixture of reticular and longitudinally oriented primary osteons. Two growth marks are visible, one very faint inner annulus and one outer annulus, which in some regions becomes a Line of Arrested Growth (LAG). Both annuli consist of relatively broad bands of parallel-fibered bone. After the second growth mark, a growth spurt is observed. This outermost region contains more strongly reticular canals, which appear larger than those in the middle and inner cortex. The animal was clearly growing rapidly at the time of death and may represent a juvenile. It is unlikely that SAM-PK-K4460 belongs to the same species as SAM-PK-K10428, SAM-PK-10188 or SAM-PK-K10000. The compact cortex is relatively thin and several thick trabeculae traverse the large medullary cavity. Several large resorption cavities and small secondary osteons are distributed around the inner cortex in places, but secondary remodeling is not extensive. A region of Sharpey's fibers extends far into the compact cortex on the posterior side.

SAM-PK-K10035 (Figure S3)

A humerus and ulna of SAM-PK-K10035 were analyzed. This specimen has been identified as *Aelurognathus tigriceps*. The bone tissue of the SAM-PK-K10035a humerus is similar to that of the SAM-PK-K4460 humerus except the vascular canal orientation is more laminar, and five growth marks of thin bands of parallel-fibered bone indicates that this individual was ontogenetically older than SAM-PK-K4460 despite being of similar size (Table 1). The trabeculae in the peri-medullary area are fragmented, thus it is difficult to determine how far they extended into the medullary cavity, but there would have been a relatively large clear area in the center of the cavity. There are few resorption cavities and secondary osteons in the inner cortex. The ulna is fragmentary from being crushed and was only used to determine the bone tissue. The vascular canals within the woven-parallel complex vary from longitudinally oriented primary osteons, simple canals, reticular to short radiating canals. Only two annuli are visible within the cortex. Both bones were still actively growing at the time of death.

SAM-PK-K10110 (Figure S3)

Although SAM-PK-K10110 has not been assigned to a species yet, it may represent a new taxon (C. Kammerer, pers. comm., 2023). The specimen includes skull material thus, it is possible it may be fully described in future. The primary cortex comprises a woven-parallel complex, but parallel-fibered bone becomes increasingly prominent towards the sub-periosteal surface in places. Three growth marks can be clearly seen, but there is possibly a fourth growth mark in the inner cortex that has been partially remodeled. In one region growth marks form LAGs and the second growth mark forms a double LAG. However, around the rest of the cross-section, the growth marks form annuli of lamellar bone, which vary from narrow bands to wide diffuse bands that appear wider than usual annuli. The outermost band reaches the sub-periosteal surface in some areas, suggesting an overall decrease in growth rate. In other places, it becomes a narrow LAG, indicating that rapid growth continued after this LAG. However, parallel-fibered bone is more prominent than woven bone in several areas of the outer cortex. This leaves open the possibility that the individual was a subadult and that the overall growth rate had decreased. The medullary cavity is relatively large, but approximately half of it is partially filled with several thick bony trabeculae. A small region in the inner cortex of the posterior side contains CCCB. Resorption cavities and secondary osteons are confined to the inner cortex.

SAM-PK-K8622 (Figure S4)

SAM-PK-K8622 includes a femur, tibia and fibula. In all these elements, the woven-parallel complex is interrupted by narrow annuli and/or LAGs. Four growth marks can be seen in each limb bone. In the fibula, the LAGs are associated with relatively wide bands of annuli comprised of parallel-fibered bone. The zone between the second and third growth mark is relatively narrower than the other zones, but there is no steady decrease in zone width towards the sub-periosteal surface, suggesting that this individual was a subadult, but still actively growing at the time of death. In the femur, thick trabeculae surrounding resorption cavities are observed in the peri-medullary cavity. Numerous small secondary osteons are distributed throughout the innermost cortex in the femur and tibia, but these features extend towards the outer cortex in one region of the tibia on the posterior side, which is associated with Sharpey’s fibers. There is also a small region of Sharpey’s fibers on the anterior side of this bone. The fibula is a thick bone with a small medullary cavity that is surrounded by a thin layer of endosteal bone. Beyond these lamellae resorption cavities, secondary osteons and CCCB surround the inner cortex almost to the mid-cortex. Sharpey’s fibers were also

observed in two regions of the fibula where they extend relatively far into the cortex on the anterior and posteromedial sides.

SAM-PK-K8623 (Figure S5)

SAM-PK-K8623 was found near SAM-PK-K8622 on the same farm. Neither specimen has been identified to species level, but it is possible they represent the same taxon. SAM-PK-K8623 is slightly larger than SAM-PK-K8622. The humerus, radius and ulna of SAM-PK-K8623 exhibit relatively thick cortices. The medullary cavities are completely infilled with bone trabeculae, with only very small clear medullary cavities in the radius and ulna. The femoral cortex is slightly thinner, but the medullary cavity is also partially infilled. A highly vascularized woven-parallel complex is present in all limb bones. Growth marks are in the form of annuli, which are so faint in some bones, they can barely be observed in normal light. The vascular orientation is reticular, but there are more radial canals in the radius. In the ulna, some parts show clear radial vascular canals between each growth mark, indicating very rapid growth. Five growth marks were observed in the humerus and radius, four in the femur and possibly six in the ulna. However, the growth marks in the femur are so faint, it is possible that the position of another growth mark could not be located. There is no indication of a decrease in growth rate towards the periphery of any of these elements and the individual was clearly still actively growing at the time of death. Sharpey's fibers were observed in the radius and femur.

SAM-PK-K10622 (Figure S6)

Two femora from specimen SAM-PK-K10622 were thin sectioned, but given their incompleteness, sections could not be taken from the middle of the midshaft. However, SAM-PK-K10622 reveals an important feature in the form of alternating zones, which differ in their vascular orientation. Although growth marks cannot be clearly observed in normal light, under cross-polarized light, there appears to be faint annuli bounding the zones that contain bone tissues with radiating vascular canals. The other zones also contain a woven-parallel complex, but the vascular canals are predominantly in the form of longitudinally oriented primary osteons transitioning into a reticular arrangement in places. Although growth marks cannot be clearly seen, there are clear cycles in growth rate in this bone.

CGS AF 391-83 (Figure S6)

A humerus, radius, ulna and femur were sectioned from a single specimen, CGS AF 391-83, which has not been identified to genus level. All elements exhibit a highly vascularized woven-parallel complex, however the amount of vascularization decreases towards the sub-periosteal surface in the humerus, radius and femur. There is a wide annulus of parallel-fibered bone tissue at the sub-periosteal surface in the ulna, so it cannot be confirmed if there was a decrease in vascularization in this element. The vascular canal arrangements vary between elements: primarily laminar in the humerus and femur, longitudinally oriented canals in the radius, and a mixture of longitudinal canals, reticular and laminar arrangements in the ulna. Five annuli comprising parallel-fibered bone were observed in the humerus and ulna. Four annuli are visible in the radius, but the section was taken further away from the middle of the midshaft compared to the other limb bones, and thus, secondary remodeling may have destroyed a few growth marks. Four growth marks could be traced in the femur. Features including the presence of several growth marks, the gradual decrease in vascularization with age and increasing proportions of parallel-fibered bone in the woven-parallel complex in parts of the outer cortex in the humerus and femur indicate an overall decrease in growth rate. This suggests that CGS AF 391-83 was a subadult, possibly even a late subadult, at the time of death. This individual would probably not have grown considerably larger as the zones become narrower towards the bone periphery. Large, thick bony trabeculae partially infill the medullary cavity of the humerus, numerous thin trabeculae completely fill the medullary cavity of the ulna, and besides a few broken fragments in the center of the femur, the medullary cavity is open. Cancellous bone is numerous in the perimedullary region of the radius, which is expected as the section was taken closer to the metaphysis of the bone where remodeling is more prominent. Secondary osteons are relatively rare, but more common in the radius, however, all are restricted to the innermost cortex. Sharpey's fibers were observed on the dorso-posterior side of the humerus, a small region on the anterior side of the radius, and two regions on the dorsal side of the femur.

CGS FL-43 (Figure S7)

CGS FL-43 is another indeterminate gorgonopsian articulated skeleton. The humerus, femur and both radii, ulnae and tibiae, were sectioned. The medullary cavities of all the elements are relatively large and clear, only broken fragments of bone have drifted into the center of the bones. Large resorption cavities are limited to the perimedullary regions and are absent from the radii. Secondary osteons are present in the inner cortex and are more prevalent in the ulnae. Secondary osteons are also more abundant in areas where there are

Sharpey's fibers, particularly in the tibiae, which have relatively extensive fibers. Thin layers of endosteal bone border the inner cortex in small areas in the humerus and radii. There is only a tiny region of endosteal bone in the femur and tibiae.

The predominant tissue type is similar to the other specimens: a woven-parallel complex. Vascular arrangements vary from mostly laminar in the humerus and femur (similar to CGS AF 391-83) to mixtures of longitudinal primary osteons and reticular canals in the radii and ulnae, with areas of a plexiform vascular orientation in the left radius. The right tibia contains mostly laminar vascular canals with some longitudinally oriented primary osteons, whereas the left tibia contains primarily longitudinal primary osteons, with lesser amounts of reticular and laminar canals. In both tibiae, long radiating vascular canals are present in areas where there are Sharpey's fibers.

A mid-cortical LAG is present on the lateral sides of the ulnae, with a second one at the sub-periosteal surface in the left ulna. A growth mark is present in both radii and the left tibia. Two growth marks were seen in the humerus. These annuli are located towards the outer cortex suggesting that the bones of this individual, preserve the first growth mark. Thus, this individual was probably in its second year of growth when it died and is likely to have been a late juvenile or early subadult. The presence of a highly vascularized woven-parallel complex all the way to the sub-periosteal surface of all the bones studied supports a relatively young ontogenetic stage. Other specimens, slightly smaller than CGS FL-43 such as SAM-PK-K8622 and SAM-PK-K8623, contain four or five growth marks, and also show no signs of decreased growth rates. Thus, it is possible this individual represents a juvenile of a larger species. Two regions on the posterior side of the humerus exhibit Sharpey's fibers that extend to the inner cortex. Sharpey's fibers were also observed on the lateral side of the right radius, and the posterior and anterior sides of both tibiae.

BP/1/4940 humerus (Figure S8)

This specimen is currently identified as an indeterminate gorgonopsian. The large medullary cavity is partially infilled by relatively thick bony trabeculae. Large resorption cavities extend into the inner cortex. Small secondary osteons lie in between the resorption cavities. The bone tissue is similar to the other specimens: a woven-parallel complex, but notably highly vascularized. Vascular canals form plexiform and reticular networks and remain abundant right up to the sub-periosteal surface. Four annuli comprised of parallel-fibered bone traverse the cortex. Under polarized light with a lambda compensator, there is a peripheral region of parallel-fibered bone. This is a relatively broad region and does not represent a growth mark,

but it is present in patches and does not follow the whole perimeter. However, some of the other annuli have similar regions before or after the actual growth mark. Thus, it is possible that a fifth annulus was starting at the sub-periosteal surface, or the outermost cortex is revealing the very beginning of a decrease in growth rate.

***Tigricephalus kingwilli* SAM-PK-K6415 (Figure S8)**

Specimen SAM-PK-K6415 was originally identified as *Arctognathus*, but it has been re-identified as *Tigricephalus kingwilli* (C. Kammerer pers. comm., 2023). Kammerer (2016) considered *Tigricephalus* a junior synonym of *Aelurognathus*, but he has recently voiced doubts about the status of *Tigricephalus*, preferring to keep these two taxa separate for now (Kammerer pers. Comm., 2023). Further study of the cranial material of this specimen will shed light on this issue. In this study, SAM-PK-K6415 is referred to *Tigricephalus* following Kammerer's latest opinion. *Tigricephalus* is similar in size to *Aelurognathus*. This specimen will be compared to SAM-PK-K10035, which has been identified as *Aelurognathus* to test how similar their bone tissues are. The radius has a relatively thick compact cortex with a small medullary cavity that is partially infilled by fragments of endosteal bone. The medullary cavity would probably have been open during life. A thin layer of endosteal bone is still in place in one part of the bone. The ulna has a relatively thinner compact cortex, but the medullary cavity is completely infilled with bony trabeculae. The inner cortex of the radius is a little fragmented, but no secondary remodeling was observed. In contrast, the ulna exhibits numerous resorption cavities and small secondary osteons in the inner cortex. Both elements exhibit similar bone tissues: a highly vascularized woven-parallel complex throughout most of the cortices. Vascular patterns in both elements tend to be reticular, but there are regions of predominantly longitudinally oriented primary osteons, and the canals become radial on the medial side of the radius in a region of prominent Sharpey's fibers. The radius reveals a clear LAG (which appears double in some places) towards the outer cortex. After this LAG the vascularization is reduced, and the bone tissue appears more parallel-fibered. This region probably represents a second growth mark and not an overall decrease in growth because the ulna was still clearly growing at the time of death. The ulna exhibits three LAGs within the outer cortex, but abundant vascular canals are seen after the third LAG, indicating that this individual had probably not even reached reproductive maturity.

The limb bones of SAM-PK-K6415 are larger than those of the *Aelurognathus* specimen SAM-PK-K10035 (Table 1). Three growth marks were observed in the former specimen, five growth marks were counted in the SAM-PK-K10035 humerus and two in the

ulna of this specimen. The radius of SAM-PK-K6415 is relatively thick and the growth marks of both elements are towards the outer cortex, suggesting that no earlier growth marks have been destroyed. This suggests that the larger SAM-PK-K6415 was ontogenetically younger than SAM-PK-K10035 and may indeed represent a different species such as *Tigricephalus*.

SAM-PK-K6407 (Figure S9)

Specimen SAM-PK-K6407 is an indeterminate gorgonopsian. A humerus and femur were thin sectioned from this individual. The humerus was not prepared from the rock matrix in order to preserve the specimen, which is relatively fragmentary. It was thin sectioned slightly below the middle of the midshaft. The cortex is relatively thin and the large medullary cavity contains numerous small bony trabeculae that almost complete fill it. Parts of the inner cortex have fragmented, but a few small secondary osteons can still be seen. The femur is less fragmentary and also contains a large medullary cavity. Resorption cavities and broken fragments of small bony trabeculae can be found in the peri-medullary area, but otherwise the medullary cavity is clear of bone. Small secondary osteons were also observed in the inner cortex. The primary bone tissue of both elements is a highly vascularized woven-parallel complex with either a laminar vascular arrangement or longitudinally oriented primary osteons. The femur contains regions of plexiform and even radial canals where Sharpey's fibers are present. Growth marks are in the form of annuli or LAGs, five were observed in the humerus and four in the femur. There is a possible sixth growth mark in the humerus, but it lies in a very small region of the innermost cortex and thus, cannot be confirmed.

BP/1/4259 (Figure S9)

A humerus, radius and ulna were analyzed from specimen BP/1/4259, which was hesitantly referred to *Cyonosaurus*, but was re-identified as an indeterminate gorgonopsian by Kammerer (pers. Comm., 2023). The humerus and radius reveal relatively thick cortices with open medullary cavities, although two thick trabeculae stretch across the medullary cavity of the humerus. The compact cortex of the ulna is relatively thinner, but the medullary cavity contains more infilling of small trabeculae and in some regions the bone wall is thicker due to the presence of compacted coarse cancellous bone. Thus, the actual open medullary cavity of this element is relatively small. A few resorption cavities and secondary osteons are present in the inner cortices of all three elements, with these features being a little more extensive in the ulna. A thin layer of endosteal bone surrounds the medullary cavity of the humerus and radius. All three bones contain a primary cortex formed predominantly of a well-vascularized

woven-parallel complex. Vascular orientations vary between the elements with a combination of laminar and longitudinally oriented primary osteons in the humerus, reticular, longitudinally oriented primary osteons and short laminar canals in the radius, and predominantly longitudinally oriented primary osteons in the ulna. Four LAGs were observed in the humerus and ulna, and three in the radius. Importantly, there does not appear to be a decrease in zone width towards the bone peripheries and the vascular canals remain abundant at the sub-periosteal surface, suggesting that the animal was still rapidly growing at the time of death. When comparing this specimen to the positively identified *Cyonosaurus* specimens SAM-PK-K10428 and SAM-PK-K10188, it is unlikely that BP/1/4259 represents *Cyonosaurus*. The latter specimen is notably larger than the *Cyonosaurus* specimens, which have several more growth marks and are thus ontogenetically older than this larger BP/1/4259 specimen. It is more likely that BP/1/4259 represents a large-bodied species of gorgonopsian.

BP/1/1258 (Figure S10)

BP/1/1258 represents a large-bodied species of indeterminate gorgonopsian. A portion of the humerus has broken away from the main body of the bone and the ulna is only partially preserved. Each bone contains a small clear medullary cavity and then bony trabeculae in the perimedullary area. Resorption cavities are abundant in the inner cortex and small secondary osteons are not particularly abundant and restricted to the innermost cortex. The humerus contains a highly vascularized woven-parallel complex with a mostly laminar vascular orientation. The radius and ulna are also well-vascularized with similar bone tissues, but they contain more longitudinally oriented primary osteons. There is a change in vascular orientation to slightly radial where there are Sharpey's fibers in the radius. Despite the relatively large size of the specimen no growth marks were observed in any of the elements. The absence of annuli or LAGs and presence of highly vascularized bone tissues at the sub-periosteal surface indicates that this animal was growing rapidly at the time of death and thus, this relatively large specimen represents a young individual of a large-bodied species.

***Arctops willistoni* BP/1/1533 (Figure S10)**

Specimen BP/1/1533 has been positively identified as *Arctops willistoni*. The large medullary cavity is filled in with small bony trabeculae and several thick bony trabeculae. Secondary osteons are abundant but are limited to the inner cortex near the resorption cavities. Most of the compact cortex comprises a highly vascularized woven-parallel complex with mostly

laminar vascular canals. Towards the outer cortex two relatively closely spaced annuli are present. The zone between these two growth marks contains more parallel-fibered bone in some regions and the outermost annulus is relatively wide. The two growth marks are not close enough to warrant identifying them as a double annulus. Thus, it appears that the growth rate of this animal was beginning to decrease at the time of death. The increasing amount of the slower forming parallel-fibered bone and smaller zone in the outer cortex suggests that this individual may have reached reproductive maturity before it died. It is the second largest limb bone and the largest humerus in the sample.

***Inostrancevia africana* NMQR4000 femur (Figure S10)**

NMQR 4000 represents a new species of South African gorgonopsian. It was recently identified as an inostrancevid, similar to the *Inostrancevia* from Russia, and has been named *Inostrancevia africana* (Kammerer et al., 2023). The medullary cavity is large and almost completely infilled with fine bony trabeculae. The compact cortex is thus, relatively thin. Secondary osteons are limited to the inner cortex. The bone tissue is like that of the other gorgonopsian species: a highly vascularized woven-parallel complex, which contains regions of laminar and reticular vascular canals as well as longitudinally oriented primary osteons. However, growth marks are absent indicating that this individual was still ontogenetically young when it died. The continued high vascularization up to the sub-periosteal surface, the absence of slower forming bone tissues and absence of growth marks strongly suggests that this animal was a juvenile. Given its large size, however, it is more likely to represent an early subadult with one or two growth marks having been destroyed by the medullary cavity expansion. It is worth noting that the entire compact cortex represents approximately 10 mm of bone growth in a single year. Despite the large size of this individual, it is clear that this animal had not reached reproductive maturity before it died and would have grown demonstrably larger had it lived to adult size.

Figures

Figure S1. Osteohistology of *Cyonosaurus* sp. SAM-PK-K10428, (a) overview of the ulna; (b) close up of ulna showing a highly vascularized WPC; (c) the ulna showing three annuli; (d) SAM-PK-10188 overview of the humerus; (e) humerus showing a peripheral region (bracket) of slower growing parallel-fibered bone; (f) humerus showing a rapidly forming WPC interrupted by four growth marks; (g) SAM-PK-10188 overview of the femur showing six growth marks; (h) WPC in the femur; (i) femur showing indistinct annuli; (j) SAM-PK-10188 overview of the radius; (k) close up of radius showing a WPC interrupted by growth marks; (l) radius showing Sharpey's fibers (arrow); (m) SAM-PK-10188 overview of the ulna; (n) close up of ulna showing five annuli; (o) SAM-PK-10188 overview of the rib showing Sharpey's fibers (arrow); (p) SAM-PK-10188 overview of the tibia showing Sharpey's fibers; (q) tibia showing a highly vascularized WPC; (r) tibia showing five annuli interrupting the bone tissue. Arrowheads indicate growth marks. Ant, anterior surface; MC, medullary cavity; med, medial surface; PFB, parallel-fibered bone; post, posterior surface; WPC, woven-parallel complex. Scale bars a, c–g = 1000 µm; b, h, i = 500 µm.

Figure S2. Osteohistology of indeterminate gorgonopsian SAM-PK-K10000, (a) overview of the humerus showing three annuli; (b) close up of humerus showing a highly vascularized WPC; (c) humerus showing three annuli and a narrow inner region of endosteal bone; (d) overview of the radius; (e) close up of radius showing a WPC interrupted by three annuli; (f) radius showing growth marks and an inner region of endosteal bone; (g) overview of the ulna; (h) ulna showing two annuli; (i) overview of tibia showing two annuli; (j) close up of tibia showing indistinct growth marks; (k) and (l) showing Sharpey's fibers (arrows) in the tibia. Arrowheads indicate growth marks. Ant, anterior surface; EB, endosteal bone; MC, medullary cavity; med, medial surface; PFB, parallel0fibered bone; post, posterior surface; WPC, woven-parallel complex. Scale bars a, d, g, i = 1000 µm; b, c, j = 500 µm; e, f, h, k, l = 100 µm.

Figure S3. Osteohistology of *Gorgonops torvus* SAM-PK-K4460, (a) overview of the humerus; (b) close up of humerus showing a peripheral growth spurt (bracket) and Sharpey's fibers (arrow); (c) humerus showing two annuli; (d) *Aelurognathus tigriceps* SAM-PK-K10035 overview of the humerus; (e) the humerus showing five annuli; (f) humerus showing a laminar vascular arrangement; (g) SAM-PK-K10035 overview of the ulna; (h) close up of

the ulna showing a WPC; (i) ulna showing two annuli; (j) SAM-PK-K10110 overview of the humerus; (k) the humerus showing two indistinct annuli (l) humerus in polarized light showing alternating regions of a WPC and PFB (brackets). Arrowheads indicate growth marks. Ant, anterior surface; lat, lateral surface; MC, medullary cavity; med, medial surface; PFB, parallel-fibered bone; post, posterior surface; WPC, woven-parallel complex. Scale bars a, d, g, j = 1000 µm; b, c, e, h, i, k, l = 500 µm; f = 100 µm.

Figure S4. Osteohistology of an indeterminate gorgonopsian SAM-PK-K8622, (a) overview of the femur; (b) close up of femur showing a WPC interrupted by five growth marks; (c) femur showing outer cortex with three LAGs; (d) overview of the tibia showing Sharpey's fibers (arrows); (e) the tibia showing a WPC; (f) tibia showing four growth marks; (g) overview of the fibula showing Sharpey's fibers (arrows); (h) close up of the fibula showing a WPC interrupted by two growth marks and Sharpey's fibers (arrow); (i) fibula showing three LAGs and an inner region of CCCB. Arrowheads indicate growth marks. Ant, anterior surface; CCCB, compacted coarse cancellous bone; lat, lateral surface; MC, medullary cavity; post, posterior surface; WPC, woven-parallel complex. Scale bars a, d, g = 1000 µm; f = 500 µm; b, c, e, h, i = 100 µm.

Figure S5. Osteohistology of an indeterminate gorgonopsian SAM-PK-K8623, (a) overview of the humerus showing an infilled medullary cavity; (b) close up of humerus showing a WPC; (c) humerus showing five annuli; (d) overview of the radius with an infilled medullary cavity and Sharpey's fibers (arrow); (e) the radius showing a WPC interrupted by four annuli; (f) radius showing a close up of Sharpey's fibers (arrow); (g) overview of the ulna showing an infilled medullary cavity; (h) close up of the ulna showing six growth marks interrupting a rapidly growing radial WPC; (i) ulna showing the radially oriented vascular canals in polarized light with a lambda compensator; (j) overview of the femur showing Sharpey's fibers (arrow); (k) femur showing a WPC interrupted by four annuli; (l) close up of the femur. Arrowheads indicate growth marks. MC, medullary cavity; R, radial vascular canals; RC, resorption cavity; WPC, woven-parallel complex. Scale bars a, d, g, j = 1000 µm; c, h, k = 500 µm; b, e, f, i, l = 100 µm.

Figure S6. Osteohistology of an indeterminate gorgonopsian SAM-PK-K10622, (a) overview of a femur; (b) close up of femur showing a WPC and a region of radiating vascular canals;

(c) femur showing two annuli; (d) overview of indeterminate gorgonopsian CGS AF 391-83 humerus; (e) close up of humerus showing an outer region of slower forming PFB; (f) humerus showing six annuli interrupting a WPC; (g) overview of the CGS AF 391-83 ulna; (h) close up of the ulna showing four annuli with the outermost one being notably thick comprised of lamellar bone; (i) overview of the CGS AF 391-83 radius; (j) close up of the radius in polarized light showing four annuli; (k) radius in polarized light with a lambda compensator showing three annuli; (l) overview of the CGS AF 391-83 femur; (m) close up of femur showing a WPC; (n) and (o) showing four annuli in the femur. Arrowheads indicate growth marks. Ant, anterior surface; MC, medullary cavity; PFB, parallel-fibered bone; post, posterior surface; R, radial vascular canals; RC, resorption cavity; WPC, woven-parallel complex. Scale bars a, d, g, h, i, l = 1000 µm; b = 500 µm; c, e, f, j, k, m, n, o = 100 µm.

Figure S7. Osteohistology of an indeterminate gorgonopsian CGS FL-43, (a) overview of the humerus showing Sharpey's fibers; (b) and (c) close up of humerus showing two annuli; (d) overview of the right ulna and radius; (e) ulna showing an annulus; (f) radius showing a WPC; (g) overview of the femur; (h) femur showing a laminar vascular arrangement; (i) femur showing a WPC in normal and polarized light with a lambda compensator; (j) overview of the right tibia; (k) right tibia showing a WPC; (l) close up of right tibia showing a WPC; (m) overview of the left ulna; (n) close up of the left ulna showing a WPC; (o) overview of the left radius; (p) overview of the left tibia showing Sharpey's fibers (arrow) and an annulus at the sub-periosteal surface; (q) close up of left tibia showing a WPC; (r) left tibia showing a growth spurt at the bone periphery. Arrowheads indicate growth marks. Ant, anterior surface; MC, medullary cavity; PFB, parallel-fibered bone; post, posterior surface; R, radial vascular canals; RC, resorption cavity; WPC, woven-parallel complex. Scale bars a, d, g, h, l = 1000 µm; b, c, e, f, i, j, k, m, n, o = 100 µm.

Figure S8. Osteohistology of indeterminate gorgonopsian BP/1/4940 and *Tigricephalus kingwilli* SAM-PK-K6415, (a) overview of the BP/1/4940 humerus; (b) BP/1/4940 showing a WPC and peripheral region of PFB; (c) BP/1/4940 showing four annuli; (d) close up of BP/1/4940 showing a WPC; (e) overview of SAM-PK-K6415 radius; (f) close up of radius showing an annulus in the outer cortex; (g) radius showing a WPC in the inner part of the cortex and peripheral PFB; (h) overview of SAM-PK-K6415 ulna; (i) close up of the ulna showing three growth marks, with the inner one represented as a double LAG; (j) ulna showing four growth marks and rapidly forming bone at the periphery. Arrowheads indicate

growth marks. Ant, anterior surface; MC, medullary cavity; med, medial; PFB, parallel-fibered bone; WPC, woven-parallel complex. Scale bars a, e, h = 1000 µm; b, c, f, g, j = 500 µm; d, i= 100 µm.

Figure S9. Osteohistology of indeterminate gorgonopsians SAM-PK-K6407 and BP/1/4259, (a) overview of SAM-PK-K6407 humerus; (b) close up of the humerus showing a WPC interrupted by five annuli, (c) a region of even more rapid bone growth between two growth marks; (d) overview of SAM-PK-K6407 femur; (e) close up of the femur showing a WPC interrupted by four annuli; (f) the femur showing thick annuli consisting of PFB; (g) overview of BP/1/4259 humerus; (h) close up of the humerus showing four growth marks with the third one represented as a double LAG; (i) close up of the humerus showing a WPC; (j) overview of BP/1/4259 radius; (k) close up of the radius showing three growth marks; (l) close up of the radius showing woven bone; (m) overview of BP/1/4259 ulna showing four growth marks; (n) close up of the ulna showing a WPC interrupted by four growth marks; (o) close up of the ulna showing a WPC. Arrowheads indicate growth marks. Ant, anterior surface; MC, medullary cavity; med, medial; PFB, parallel-fibered bone; post, posterior; WPC, woven-parallel complex. Scale bars a, d, g, j, m = 1000 µm; b, c, e, f, h, k, n, o = 500 µm; i, l= 100 µm.

Figure S10. Osteohistology of indeterminate gorgonopsian BP/1/1258, *Arctops willistoni* BP/1/1533 and *Inostrancevia africana* NMQR 4000, (a) overview of BP/1/1258 humerus; (b) and (c) close up of the humerus showing a WPC in polarized light with a lambda compensator and in normal light; (d) overview of BP/1/1258 radius; (e) and (f) close up of radius showing a WPC in polarized light and polarized light with a lambda compensator; (g) overview of BP/1/1258 ulna; (h) and (i) showing a WPC; (j) overview of *Arctops willistoni* BP/1/1533 humerus; (k) close up of humerus showing a region of WPC and an outer region of PFB with a growth mark at the sub-periosteal surface; (l) humerus showing increased PFB with a wide outer region of lamellar bone; (m) outer region of PFB; (n) overview of *Inostrancevia africana* NMQR 4000 femur; (o) femur showing a WPC; (p) close up of femur showing a WPC. Note the white line that appears to represent an annulus, however, this is the only region of the bone that exhibits this feature, indicating that this does not represent a growth mark. Arrowheads indicate growth marks. LB, lamellar bone; MC, medullary cavity;

PFB, parallel-fibered bone; WPC, woven-parallel complex. Scale bars a, d, j, n = 5000 μm; g = 1000 μm; b, c, e, f, k, l, o = 500 μm; h, i, m, p = 100 μm.
