# Supplementary material for: The osteohistology of gorgonopsian therapsids and implications for Permo‐Triassic theriodont growth
Source: J Anat. 2024 Dec 20;246(6):987–1000. doi: 10.1111/joa.14201 (PMC12079768; doi:10.1111/joa.14201)
Supplement: Supplementary file 12 — Data S2. [file JOA-246-987-s008.pdf]

## Supplementary Information 2

Table S1. Zone widths between each growth mark for each element in this study.

| Accession number | Identification | Element | Area mm$^2$ | C (mm) | GM | MC to GM1 | to GM2 |
|---|---|---|---|---|---|---|---|
| | **Gorgonopsians** | | | | | | |
| SAM-PK-K10428b | *Cyonosaurus* | ulna | — | — | 2 | — | — |
| SAM-PK-10188a | *Cyonosaurus* | humerus | — | — | 6 | — | — |
| SAM-PK-10188b | *Cyonosaurus* | femur | 134.98R | 41.2 | 6 | 1259.15 | 904.06 |
| SAM-PK-10188c | *Cyonosaurus* | radius | 38.66 | 22.04 | 5 | 643.91 | 386.26 |
| SAM-PK-10188d | *Cyonosaurus* | ulna | 55.15 | 26.32 | 5 | 1669.83 | 1199.84 |
| SAM-PK-10188e | *Cyonosaurus* | tibia | 71.27 | 29.93 | 6 | 1367.44 | 823.62 |
| SAM-PK-10188f | *Cyonosaurus* | rib | 27.5 | 18.59 | 5 | 448.71 | 328.32 |
| SAM-PK-K10000a | indeterminate | humerus | 384.61 | 69.52 | 2 | 4378.92 | 860.11 |
| SAM-PK-K10000b | indeterminate | radius | 45.29 | 23.86 | 3 | 610.87 | 1051.75 |
| SAM-PK-K10000c | indeterminate | ulna | 42.35 | 23.07 | 2 | 544.38 | 1062.81 |
| SAM-PK-K10000d | indeterminate | tibia | 92.8 | 34.15 | 2 | 218.9 | 773.98 |
| SAM-PK-K4460 | *Gorgonops torvus* | humerus | 215.53 | 52.04 | 2 | 2341.83 | 1151.85 |
| SAM-PK-K10035a | *Aelurognathus tigriceps* | humerus | 222.51 | 52.88 | 5 | 686.41 | 286.28 |
| SAM-PK-K10035b | *Aelurognathus tigriceps* | ulna | — | — | 2 | — | — |
| SAM-PK-K10110 | possible new species | humerus | 223.06 | 52.94 | 3 | 1332.66 | 536.83 |
| SAM-PK-K8622a | indeterminate | femur | 177.09 | 47.17 | 4 | 368.77 | 1327.5 |
| SAM-PK-K8622b | indeterminate | tibia | 113.14 | 37.71 | 4 | 1455.03 | 1094.19 |
| SAM-PK-K8622c | indeterminate | fibula | 49.96 | 25.06 | 4 | 642.69 | 1192.85 |
| SAM-PK-K8623a | indeterminate | humerus | 291.03 | 60.47 | 6 | 580.5 | 706.99 |
| SAM-PK-K8623b | indeterminate | ulna | 134.33 | 41.09 | 6 | 629.61 | 310.84 |
| SAM-PK-K8623c | indeterminate | radius | 115.36 | 38.07 | 5 | 2402.43 | 693.32 |
| SAM-PK-K8623d | indeterminate | femur | 227.62 | 53.48 | 4 | 2975.04 | 626.85 |
| SAM-PK-K10622c | indeterminate | femur | — | — | 4 | — | — |
| SAM-PK-K10622d | indeterminate | femur | — | — | 2 | — | — |

| | | | | | | | |
|---|---|---|---|---|---|---|---|
| CGS AF 391-83a | indeterminate | humerus | 292.42 | 60.62R | 6 | 641.89 | 945.59 |
| CGS AF 391-83b | indeterminate | radius | — | — | 4 | — | — |
| CGS AF 391-83c | indeterminate | ulna | 91.17 | — | 5 | 1114.85 | 285.02 |
| CGS AF 391-83d | indeterminate | femur | 272.13 | 58.48 | 4 | 1752.26 | 399.32 |
| CGS FL-43a | indeterminate | humerus | 284.55R | 59.8 | 2 | 2935.36 | 487.21 |
| CGS FL-43b | indeterminate | radius right | 134.72 | 41 | 1 | 2956.9 | 565.82 |
| CGS FL-43c | indeterminate | ulna right | 161.65 | 45.07 | 1 | 5760.67 | — |
| CGS FL-43d | indeterminate | femur | 351.04 | 66.42 | 0 | 5935.1 | — |
| CGS FL-43e | indeterminate | tibia right | 276.55 | 59 | 0 | 5778.37 | — |
| CGS FL-43f | indeterminate | ulna left | 153.15 | 43.87 | 2 | 1082.72 | 447.43 |
| CGS FL-43g | indeterminate | radius left | 126.55 | 39.88 | 1 | 5235.74 | 474.75 |
| CGS FL-43h | indeterminate | tibia left | 254.18 | 56.52 | 1? | 5467.46 | — |
| BP/1/4940 | indeterminate | humerus | 319.15 | 63.33 | 5 | 1416.5 | 1047.97 |
| SAM-PK-K6415a | *Tigricephalus kingwilli* | radius | 198.97 | 50 | 2 | 3363.47 | 797.89 |
| SAM-PK-K6415b | *Tigricephalus kingwilli* | ulna | 316.5 | 63.07 | 3 | 4384.56 | 1095.53 |
| SAM-PK-K6407a | indeterminate | humerus | 489.08 | 78.4 | 5-6 | 843.96 | 733.96 |
| SAM-PK-K6407b | indeterminate | femur | 706.9 | 94.25 | 4 | 1882.21 | 1513.72 |
| BP/1/4259a | indeterminate | humerus | 566.43 | 84.37 | 4 | 2826.82 | 973.03 |
| BP/1/4259b | indeterminate | radius | 229.75 | 53.73 | 3 | 3613.68 | 755.31 |
| BP/1/4259c | indeterminate | ulna | 231.12 | 53.89 | 4 | 2856.84 | 486.3 |
| BP/1/1258a | indeterminate | humerus | 537.42 | 82.18 | 0 | 9628.81 | — |
| BP/1/1258b | indeterminate | radius | 293.75 | 60.76 | 0 | 7168.98 | — |
| BP/1/1258c | indeterminate | ulna | 261.97 | 57.38 | 0 | 7942.8 | — |
| BP/1/1533 | *Arctops willistoni* | humerus | 1269.58 | 126.31 | 2 | 15092.7 | 2055.41 |
| NMQR 4000 | *Inostrancevia africana* | femur | 1388.09 | 132.07 | 0 | 10445.81 | — |
| | **Therocephalians** | | | | | | |
| SAM-PK-K9084 | *Lycosuchus vanderrieti* | ulna | 345.84 | 65.92 | 4 | 3774.75 | 1646.77 |
| BP/1/5587a | scylacosaurid | humerus | 203.24 | 50.55 | 2 | 1753.16 | 1851.24 |

| | | | | | | | |
|---|---|---|---|---|---|---|---|
| BP/1/5587b | scylacosaurid | ulna | 116.96R | 38.34R | 5 | 3218.92 | 1349.25 |
| BP/1/4227 | *Moschorhinus kitchingi* | humerus | 323.16 | 63.73 | 3 | 1732.67 | 1773.74 |
| NMQR3939a | *Moschorhinus kitchingi* | humerus | 435.54 | 74 | 3 | 6168.76 | 1200.18 |
| NMQR3939b | *Moschorhinus kitchingi* | radius | 74.49 | 31 | 3 | 2264.3 | 361.84 |
| NMQR3939c | *Moschorhinus kitchingi* | tibia | 115.67 | 38.13 | 3 | 2846.03 | 833.54 |
| NMQR3684 | *Moschorhinus kitchingi* | femur | 432.84 | 73.75 | 0 | 9698.96 | — |
| | **Non-mammaliaform cynodonts** | | | | | | |
| SAM-PK-6235 | *Cynognathus crateronotus* | femur | 343.52 | 65.7 | 0 | 7858.3 | — |
| NMQR1208a | *Cynognathus crateronotus* | humerus | 313.65 | 62.78 | 0 | 6182.46 | — |
| NMQR1208f | *Diademodon tetragonus* | femur | 267.76 | 58 | 4 | 4513.66 | 1038.92 |
| NMQR1208c | *Diademodon tetragonus* | humerus | 395.12 | 70.46 | 5 | 5599.95 | 1169.92 |

Table S1. Continued: from the third growth mark to the last growth mark of each element.

| Accession number | Identification | Element | to GM3 | to GM4 | to GM5 | to GM6 | to periphery |
|---|---|---|---|---|---|---|---|
| | **Gorgonopsians** | | | | | | |
| SAM-PK-K10428b | *Cyonosaurus* | ulna | — | — | — | — | — |
| SAM-PK-10188a | *Cyonosaurus* | humerus | — | — | — | — | — |
| SAM-PK-10188b | *Cyonosaurus* | femur | 540.62 | 404.01 | 337.51 | | — |
| SAM-PK-10188c | *Cyonosaurus* | radius | 389.85 | 368.01 | 265.24 | 269.48 | — |
| SAM-PK-10188d | *Cyonosaurus* | ulna | 585.88 | 401.01 | 947.52 | 497.86 | — |
| SAM-PK-10188e | *Cyonosaurus* | tibia | 481.98 | 422.8 | 341.19 | 298.16 | — |
| SAM-PK-10188f | *Cyonosaurus* | rib | 192.01 | 139.68 | 132.63 | 187.4 | — |
| SAM-PK-K10000a | indeterminate | humerus | 166.92 | — | — | — | — |
| SAM-PK-K10000b | indeterminate | radius | 549.65 | 426.42 | — | — | — |
| SAM-PK-K10000c | indeterminate | ulna | 846.44 | — | — | — | — |
| SAM-PK-K10000d | indeterminate | tibia | 719.43 | — | — | — | — |
| SAM-PK-K4460 | *Gorgonops torvus* | humerus | 619.26 | — | — | — | — |
| SAM-PK-K10035a | *Aelurognathus tigriceps* | humerus | 546.12 | 738.49 | 384.91 | 103.23 | — |
| SAM-PK-K10035b | *Aelurognathus tigriceps* | ulna | — | — | — | — | — |
| SAM-PK-K10110 | possible new species | humerus | 1659.84 | 1780.09 | | — | — |
| SAM-PK-K8622a | indeterminate | femur | 481.73 | 651.91 | 292.68 | — | — |
| SAM-PK-K8622b | indeterminate | tibia | 528.07 | 1746.93 | 313.37 | — | — |
| SAM-PK-K8622c | indeterminate | fibula | 351.92 | 498.91 | 350.06 | — | — |
| SAM-PK-K8623a | indeterminate | humerus | 867.9 | 655.53 | 697.05 | 407.19 | 583.46 |
| SAM-PK-K8623b | indeterminate | ulna | 273.45 | 137.31 | 336.25 | 482.25 | 251.02 |
| SAM-PK-K8623c | indeterminate | radius | 1081.44 | 376.8 | 588.17 | 379.38 | 488.16 |
| SAM-PK-K8623d | indeterminate | femur | 717.96 | 514.12 | 1157.29 | — | — |
| SAM-PK-K10622c | indeterminate | femur | — | — | — | — | — |
| SAM-PK-K10622d | indeterminate | femur | — | — | — | — | — |
| CGS AF 391-83a | indeterminate | humerus | 567.62 | 591.2 | 284.84 | 294.17 | — |

| | | | | | | | |
|---|---|---|---|---|---|---|---|
| CGS AF 391-83b | indeterminate | radius | — | — | — | — | — |
| CGS AF 391-83c | indeterminate | ulna | 406.48 | 986.77 | 378.36 | 66.53 | — |
| CGS AF 391-83d | indeterminate | femur | 399.32 | 307.59 | 57.32 | — | — |
| CGS FL-43a | indeterminate | humerus | 280.79 | — | — | — | — |
| CGS FL-43b | indeterminate | radius right | — | — | — | — | — |
| CGS FL-43c | indeterminate | ulna right | — | — | — | — | — |
| CGS FL-43d | indeterminate | femur | — | — | — | — | — |
| CGS FL-43e | indeterminate | tibia right | — | — | — | — | — |
| CGS FL-43f | indeterminate | ulna left | 63.41 | — | — | — | — |
| CGS FL-43g | indeterminate | radius left | — | — | — | — | — |
| CGS FL-43h | indeterminate | tibia left | — | — | — | — | — |
| BP/1/4940 | indeterminate | humerus | 982.31 | 958.18 | 720.25 | 1698.4 | — |
| SAM-PK-K6415a | *Tigricephalus kingwilli* | radius | 75.96 | — | — | — | — |
| SAM-PK-K6415b | *Tigricephalus kingwilli* | ulna | 767.05 | 182.82 | — | — | — |
| SAM-PK-K6407a | indeterminate | humerus | 944.46 | 2620.3 | 1555.53 | 481.55 | — |
| SAM-PK-K6407b | indeterminate | femur | 3631.44 | 1381.27 | 535.36 | — | — |
| BP/1/4259a | indeterminate | humerus | 893.82 | 868.79 | — | — | — |
| BP/1/4259b | indeterminate | radius | 1032.29 | 1319.54 | — | — | — |
| BP/1/4259c | indeterminate | ulna | 1306.76 | 753.05 | — | — | — |
| BP/1/1258a | indeterminate | humerus | — | — | — | — | — |
| BP/1/1258b | indeterminate | radius | — | — | — | — | — |
| BP/1/1258c | indeterminate | ulna | — | — | — | — | — |
| BP/1/1533 | *Arctops willistoni* | humerus | 403.22 | — | — | — | — |
| NMQR 4000 | *Inostrancevia africana* | femur | — | — | — | — | — |
| | **Therocephalians** | | | | | | |
| SAM-PK-K9084 | *Lycosuchus vanderrieti* | ulna | 2058.99 | 1107.9 | 582.7 | — | — |
| BP/1/5587a | scylacosaurid | humerus | 1520.08 | — | — | — | — |
| BP/1/5587b | scylacosaurid | ulna | 1247.96 | 2086.8 | 908.86 | 553.34 | — |
| BP/1/4227 | *Moschorhinus kitchingi* | humerus | 2369.1 | 295.92 | — | — | — |

| | | | | | | | |
|---|---|---|---|---|---|---|---|
| NMQR3939a | *Moschorhinus kitchingi* | humerus | 1272.87 | 1151.81 | — | — | — |
| NMQR3939b | *Moschorhinus kitchingi* | radius | 440.23 | 388.79 | — | — | — |
| NMQR3939c | *Moschorhinus kitchingi* | tibia | 871.67 | 586.54 | — | — | — |
| NMQR3684 | *Moschorhinus kitchingi* | femur | — | — | — | — | — |
| | **Non-mammaliaform cynodonts** | | | | | | |
| SAM-PK-6235 | *Cynognathus crateronotus* | femur | — | — | — | — | — |
| NMQR1208a | *Cynognathus crateronotus* | humerus | — | — | — | — | — |
| NMQR1208f | *Diademodon tetragonus* | femur | 1160.79 | 1005.34 | 551.34 | — | — |
| NMQR1208c | *Diademodon tetragonus* | humerus | 611.1 | 1678.02 | 978.78 | 1097.29 | — |

Table S2. Cortical thickness values of the gorgonopsians, therocephalians and non-mammaliform cynodonts used in this study (see legend below for abbreviations).

| Identification | Accession number | Element | Min | Max | Cg | CDI | S | P |
|---|---|---|---|---|---|---|---|---|
| **Gorgonopsians** | | | | | | | | |
| *Cyonosaurus* | SAM-PK-10188c | radius | 0.078 | 0.999 | 0.894 | 0.683 | 0.062 | 0.317 |
| *Cyonosaurus* | SAM-PK-10188d | ulna | 0.247 | 0.999 | 0.910 | 0.666 | 0.045 | 0.334 |
| *Cyonosaurus* | SAM-PK-10188e | tibia | 6.81E-07 | 0.999 | 0.926 | 0.732 | 0.019 | 0.267 |
| indeterminate | SAM-PK-K10000a | humerus | 0.142 | 0.999 | 0.676 | 0.399 | 0.061 | 0.601 |
| indeterminate | SAM-PK-K10000b | radius | 6.697E-07 | 0.999 | 0.776 | 0.532 | 0.029 | 0.468 |
| indeterminate | SAM-PK-K10000c | ulna | 6.230E-07 | 0.999 | 0.711 | 0.471 | 0.043 | 0.529 |
| indeterminate | SAM-PK-K10000d | tibia | 3.268E-07 | 0.999 | 0.662 | 0.424 | 0.027 | 0.576 |
| *Gorgonops torvus* | SAM-PK-K4460 | humerus | 1.101E-06 | 0.999 | 0.808 | 0.593 | 0.086 | 0.407 |
| new species? | SAM-PK-K10110 | humerus | 0.482 | 0.999 | 0.809 | 0.415 | 0.085 | 0.584 |
| indeterminate | SAM-PK-K8622a | femur | 3.855E-07 | 0.999 | 0.666 | 0.432 | 0.051 | 0.568 |
| indeterminate | SAM-PK-K8622b | tibia | 0.0140 | 0.999 | 0.867 | 0.643 | 0.039 | 0.357 |
| indeterminate | SAM-PK-K8622c | fibula | 6.85E-07 | 0.999 | 0.869 | 0.654 | 0.056 | 0.346 |
| indeterminate | SAM-PK-K8623a | humerus | 0.563 | 0.999 | 0.915 | 0.567 | 0.041 | 0.433 |
| indeterminate | SAM-PK-K8623b | ulna | 0.095 | 0.999 | 0.943 | 0.801 | 0.086 | 0.199 |
| indeterminate | SAM-PK-K8623c | radius | 0.427 | 0.999 | 0.934 | 0.673 | 0.045 | 0.327 |
| indeterminate | SAM-PK-K8623d | femur | 6.847E-07 | 0.999 | 0.808 | 0.568 | 0.032 | 0.431 |
| indeterminate | CGS AF 391-83a | humerus | 6.847E-07 | 0.999 | 0.649 | 0.417 | 0.051 | 0.583 |
| indeterminate | CGS AF 391-83c | ulna | 0.428 | 0.999 | 0.871 | 0.535 | 0.048 | 0.464 |
| indeterminate | CGS FL-43b | radius right | 6.848E-07 | 0.999 | 0.830 | 0.596 | 0.038 | 0.404 |
| indeterminate | CGS FL-43c | ulna right | 0.109 | 0.999 | 0.818 | 0.556 | 0.039 | 0.444 |
| indeterminate | CGS FL-43d | femur | 0.079 | 0.999 | 0.712 | 0.450 | 0.049 | 0.550 |
| indeterminate | CGS FL-43e | tibia right | 0.282 | 0.999 | 0.843 | 0.555 | 0.076 | 0.444 |
| indeterminate | CGS FL-43f | ulna left | 0.329 | 0.999 | 0.848 | 0.536 | 0.050 | 0.464 |
| indeterminate | CGS FL-43g | radius left | 0.111 | 0.999 | 0.826 | 0.563 | 0.032 | 0.436 |
| indeterminate | CGS FL-43h | tibia left | 0.013 | 0.999 | 0.756 | 0.511 | 0.041 | 0.489 |

| | | | | | | | | |
|---|---|---|---|---|---|---|---|---|
| indeterminate | BP/1/4940 | humerus | 0.098 | 0.999 | 0.804 | 0.560 | 0.082 | 0.439 |
| *Tigricephalus kingwilli* | SAM-PK-K6415a | radius | 0.270 | 0.999 | 0.817 | 0.505 | 0.033 | 0.495 |
| *Tigricephalus kingwilli* | SAM-PK-K6415b | ulna | 2.293E-06 | 0.999 | 0.899 | 0.713 | 0.074 | 0.287 |
| indeterminate | SAM-PK-K6407b | femur | 0.009 | 0.999 | 0.745 | 0.501 | 0.044 | 0.498 |
| indeterminate | BP/1/4259a | humerus | 0.262 | 0.999 | 0.835 | 0.534 | 0.032 | 0.466 |
| indeterminate | BP/1/4259b | radius | 6.830E-07 | 0.999 | 0.827 | 0.592 | 0.039 | 0.408 |
| indeterminate | BP/1/1258a | humerus | 6.739E-07 | 0.999 | 0.814 | 0.586 | 0.060 | 0.414 |
| indeterminate | BP/1/1258b | radius | 0.039 | 0.999 | 0.848 | 0.613 | 0.045 | 0.386 |
| *Arctops willistoni* | BP/1/1533 | humerus | 0.492 | 0.999 | 0.861 | 0.490 | 0.057 | 0.509 |
| *Inostrancevia africana* | NMQR 4000 | femur | 0.158 | 0.999 | 0.687 | 0.408 | 0.073 | 0.592 |
| **Therocephalians** | | | | | | | | |
| *Lycosuchus vanderrieti* | SAM-PK-K9084 | ulna | 0.238 | 0.999 | 0.892 | 0.634 | 0.043 | 0.366 |
| scylacosaurid | BP/1/5587a | humerus | 0.212 | 0.999 | 0.898 | 0.660 | 0.062 | 0.340 |
| scylacosaurid | BP/1/5587b | ulna | 1.691E-06 | 0.999 | 0.953 | 0.802 | 0.047 | 0.198 |
| *Moschorhinus kitchingi* | BP/1/4227 | humerus | 0.242 | 0.999 | 0.904 | 0.656 | 0.043 | 0.344 |
| *Moschorhinus kitchingi* | NMQR3939a | humerus | 0.482 | 0.999 | 0.8940 | 0.557 | 0.036 | 0.443 |
| *Moschorhinus kitchingi* | NMQR3939b | radius | 1.279E-06 | 0.999 | 0.976 | 0.913 | 0.077 | 0.087 |
| *Moschorhinus kitchingi* | NMQR3939c | tibia | 1.274E-06 | 0.999 | 0.954 | 0.828 | 0.069 | 0.173 |
| *Moschorhinus kitchingi* | NMQR3684d | femur | 6.848E-07 | 0.999 | 0.932 | 0.787 | 0.083 | 0.213 |
| **Non-mammaliaform cynodonts** | | | | | | | | |
| *Cynognathus crateronotus* | SAM-PK-6235 | femur | 0.056 | 0.999 | 0.912 | 0.703 | 0.034 | 0.297 |
| *Cynognathus crateronotus* | NMQR1208a | humerus | 0.371 | 0.999 | 0.831 | 0.490 | 0.036 | 0.510 |
| *Diademodon tetragonus* | NMQR1208f | femur | 1.271E-06 | 0.999 | 0.912 | 0.712 | 0.033 | 0.288 |
| *Diademodon tetragonus* | NMQR1208c | humerus | 0.001 | 0.999 | 0.909 | 0.721 | 0.061 | 0.279 |

**Legend**

| | |
|---|---|
| **Min** | Lower asymptote, compactness in the middle of the medullary cavity |
| **Max** | Upper asymptote, compactness in the outermost cortex |
| **Cg** | Global compactness, i.e. the entire section of bone |
| **CDI** | Cortico-diaphyseal index (cortical thickness) |
| **S** | Width of the cortico-medullary transition zone |
| **P** | Extent of medullary cavity |
